# Use of dignity therapy in palliative care: a comprehensive scoping review

**DOI:** 10.1186/s12904-025-01812-4

**Published:** 2025-07-01

**Authors:** Romel Jonathan Velasco Yanez, Erilaine de Freitas Corpes, Judith Sixsmith, Ana Fátima Carvalho Fernandes, Priscila de Souza Aquino, Régia Christina Moura Barbosa Castro, Herla Maria Furtardo Jorge

**Affiliations:** 1 Department of Nursing, Federal University of Ceará, Fortaleza, Brazil; 2 School of Health Sciences, University of Dundee, 11 Airlie Pl, Dundee DD1 4HJ, Dundeee, Scotland, UK

**Keywords:** Dignity, Dignity therapy, Palliative care, End-of-life care, Scoping review

## Abstract

**Introduction:**

Dignity Therapy is an innovative intervention designed to alleviate emotional suffering and address the distress associated with loss of dignity in individuals with advanced illness or at the end of life. Its use has steadily increased, and previous systematic reviews have synthesized evidence on its effectiveness in palliative care. However, there is no comprehensive synthesis that explores aspects of this intervention beyond commonly reported clinical outcomes. The aim of this review was to identify and synthesize the available evidence on the use of Dignity Therapy in palliative care.

**Methods:**

This comprehensive scoping review followed the Joanna Briggs Institute methodology, and the final report was prepared in accordance with the PRISMA-ScR checklist. The protocol was registered on the Open Science Framework (DOI: 10.17605/OSF.IO/MNWUJ). From 2016 onward, systematic searches were conducted in 11 databases, including gray literature. Study selection was performed using RAYYAN software, and qualitative content analysis was used for data analysis.

**Results:**

Of the 815 records identified, 82 articles with 11 Gy literature documents included. Most studies were published in 2023 (19.5%), originated from the United States (23.1%), used an experimental design (29.2%), and focused on oncology (58.5%). Nine key categories emerged from the analysis: feasibility, acceptability, satisfaction, and effectiveness; perceived health benefits; impact on family; adaptation to diverse contexts; use of technology; economic feasibility; professional profiles; and implementation in underrepresented populations.

**Conclusions:**

The evidence suggests benefits across various dimensions of health, including among family members, pediatric and adolescent populations, and traditionally underrepresented groups, as well as its implementation in early stages of illness and posthumous delivery. The expansion of Dignity Therapy has also driven cultural and linguistic adaptations, the development of derivative tools, and its application in a range of clinical contexts, along with the involvement of a broader spectrum of professionals. This review provides a comprehensive synthesis to guide healthcare professionals on the current state of knowledge, while identifying key research gaps and new insights to inform future studies.

**Supplementary Information:**

The online version contains supplementary material available at 10.1186/s12904-025-01812-4.

## Introduction

Palliative care is an approach aimed at improving the quality of life for patients and their families as they face health challenges associated with life-threatening illnesses. The goal of palliative care is to prevent and alleviate suffering through the early identification, appropriate assessment and treatment of pain and other issues, whether they are physical, psychosocial, or spiritual [1]. It is estimated that over 56.8 million people worldwide require palliative care each year, including 25.7 million who are nearing the end of life, yet only 14% of those in need receive this support [2–4].

Currently, palliative care is at the forefront of discussions among major health organizations due to the potential benefits its early implementation can offer, especially in countries with significant disparities in access to chronic disease care [4]. In this context, advancements in the field have progressively facilitated its integration into healthcare systems, with the purpose of addressing the care needs of patients from the moment they are diagnosed with life-threatening illnesses, alongside their active treatment. This progress has driven the development of various therapies designed to provide cost-effective tools that enhance quality of life and meet the specific care needs of patients receiving palliative care [5].

As an example of these interventions, in 2002 Harvey Chochinov [6] developed Dignity Therapy, a brief and individualized psychotherapy designed to alleviate the psycho-emotional and existential distress of patients with advanced illnesses and limited life expectancy. Dignity Therapy provides patients with the opportunity to reflect on meaningful matters, values, achievements, or messages they wish to convey to others [6, 7]. This intervention is structured around guided interviews using the Dignity Therapy Question Protocol, where patients share and reflect on significant aspects of their lives. Their responses are recorded, transcribed, and compiled into a legacy document. This document is intended to preserve the patient’s dignity, strengthen emotional bonds, and provide a meaningful keepsake for their loved ones [8].

A quick search of major health databases revealed evidence suggesting significant benefits of Dignity Therapy [9–12]. However, some studies have also reported inconclusive findings regarding its actual impact [13–16]. Moreover, although several reviews on Dignity Therapy have been published in recent years [17–19], their methodological approaches —effectiveness reviews— may have limited the inclusion of other study designs that could provide a more comprehensive understanding of this therapy. For this reason, this study draws on the review conducted by Martínez et al. [7], which thoroughly examined studies on Dignity Therapy in palliative care from 2002 to 2016. According to the authors’ knowledge, this was the first published review on the topic.

Finally, this review will establish the state of the art for developing a randomized clinical trial on the use of Dignity Therapy in cancer patients in Brazil, which will be the first known clinical trial conducted in Latin America within this context. Based on the considerations outlined above, the objective of this review is to identify and synthesize the existing evidence on the use of Dignity Therapy in palliative care.

## Methods

This is a scoping review guided by the methodological recommendations of the Joanna Briggs Institute (JBI) [20]. The final report was written following the PRISMA Extension for Scoping Reviews (PRISMA-ScR) checklist [21] (Supplementary material 1), and the review protocol was registered on the Open Science Framework website under DOI no. 10.17605/OSF.IO/MNWUJ.

### Review question

The review question was developed using the Population, Concept, Context (PCC) strategy proposed by the JBI [20]. In this case, Dignity Therapy was identified as the concept, and palliative care formed the context. Based on this, the following review question was formulated: What evidence exists on the use of Dignity Therapy in palliative care? It is important to note that no specific population was defined. While this review will establish the foundation for a randomized control trial in adult cancer patients, the authors considered it essential to include all age groups with palliative care needs to ensure the findings can support researchers working with Dignity Therapy across diverse age-related contexts.

### Eligibility criteria

All studies addressing the variables outlined in the PCC acronym were included, without restrictions on methodological design. The review was limited to studies published in Spanish, English, or Portuguese from January 2016 onward [7].

### Inclusion criteria

#### Concept

All studies that applied Dignity Therapy using the original protocol proposed by Chochinov [6, 7] were included. Additionally, studies that adapted Dignity Therapy for application in other contexts were also included.

**Context** All studies addressing palliative care were included, based on the definition provided by the World Health Organization (WHO): “Palliative care improves the quality of life of patients and that of their families who are facing challenges associated with life-threatening illness, whether physical, psychological, social or spiritual” [22]. It is important to note that in recent years, various terms related to palliative care, such as *“end-of-life care”* or *“terminal care”* [23], have emerged and are often used interchangeably with *“palliative care”*. Since Dignity Therapy was originally designed for application in end-of-life contexts, including these terms in the review was considered relevant to capture studies using such terminology. However, based on existing literature and our knowledge, these terms are integral to the broader philosophy of palliative care and are applied during specific phases and contexts along the natural trajectory of the disease. Therefore, they should not be studied or applied in isolation.

### Exclusion criteria

Studies focused exclusively on secondary qualitative analyses of legacy documents produced as part of Dignity Therapy interventions were excluded. While such studies may offer valuable interpretations, they do not provide detailed information on the primary outcomes of the original research, and therefore do not align with the objective of this review. Similarly, records of randomized controlled trial protocols were excluded when full study results were already available. Reviews that did not contribute new information and whose findings were based solely on primary studies already included in our analysis were also excluded, in accordance with methodological recommendations aimed at avoiding double counting.

### Search strategy

The search strategy was developed following the recommendations of the JBI [20]. Initially, a limited search was conducted in the Medline/PubMed, EMBASE and LILACS databases to locate relevant studies on the topic. This strategy included key descriptors associated with the PCC framework ((“Palliative care” [mesh terms] OR “End of Life Care” [tiab]) AND (“Dignity Therapy” [mesh terms])). Next, the articles retrieved during this preliminary search were reviewed by the lead author, who analyzed titles and abstracts to identify free terms commonly used in studies on the topic. These free terms were then supplemented with descriptors and synonyms drawn from controlled vocabularies (MeSH, DeCS, Emtree, and CINAHL Headings). The terms were combined using the boolean operators “AND” and “OR” to create tailored search strategies for the following databases: Medline (via PubMed), EMBASE, Cochrane Library, Web of Science, Scopus, Epistemonikos, CINAHL, APA PsycInfo, Latin America and the Caribbean Literature on Health Sciences (LILACS), Banco de dados da Enfermagem *[Nursing Database]* (BDENF), and the Índice Bibliográfico Español de Ciencias de la Salud *[Spanish Bibliographic Index of Health Sciences]* (IBECS) (Supplementary material 2). Lastly, reverse searches were also conducted on the reference lists of all included articles to identify further studies relevant to the final sample.

Secondary searches for gray literature were conducted using the sources Google Scholar, OpenGrey, the Institutional Repository for Information Sharing (WHO Database), and ProQuest Global Dissertations and Theses. In addition, the websites of scientific societies, including the American Academy of Hospice and Palliative Medicine (AAHPM) and the European Association for Palliative Care (EAPC), were explored. In cases where articles were not electronically accessible and required a full review, they were requested directly from the authors via ResearchGate (*n* = 4). It is important to note that a date filter (from 2016 onward) was applied only in databases that allowed it.

All searches, including those for peer-reviewed and gray literature, were conducted on November 6, 2023. However, due to the time elapsed since the initial search and the growing use of Dignity Therapy, the peer-reviewed literature searches were updated on April 24, 2025.

### Source of evidence selection

The management of the retrieved sources of evidence was conducted using the software Rayyan Systems Inc (RAYYAN) [24]. Initially, all articles obtained from the general search were uploaded to RAYYAN, where duplicate studies were identified and removed. To ensure that selection decisions were not influenced by the subjective interpretations of a single reviewer, the agreement rate between two reviewers was verified to be ≥ 75%, in accordance with JBI guidelines [20]. To achieve this, two reviewers analyzed the titles and abstracts of an initial sample of 25 articles, and after this preliminary review, they met to discuss any selection discrepancies, achieving an agreement rate of 72%. This result required an additional meeting between the reviewers to verify and confirm the predefined eligibility criteria. A second sample of 25 articles was then evaluated, resulting in a final agreement rate of 96%.

Once this phase was completed, the two reviewers analyzed all studies by title and abstract to identify potentially eligible articles based on the inclusion and exclusion criteria. The selected articles were then reviewed in full by the same reviewers to compile a final list of studies for inclusion. It is important to note that readings were conducted independently and blindly. Additionally, throughout each stage of this process, the reviewers held regular meetings to discuss any decision conflicts, and any discrepancies were resolved by a third reviewer. This same procedure was maintained during the study selection phase of the updated search.

The selection of gray literature was conducted exclusively by the main author, who manually reviewed the documents identified in secondary searches based on the pre-established eligibility criteria. Once selected, these documents were included in the final sample. A detailed outline of the entire procedure is provided in the PRISMA 2020 flow diagram [25].

### Data extraction

Data extraction was conducted independently and in a blinded manner by the same reviewers [20]. The data extraction sheet was adapted from other studies [26, 27] and included the following variables: *(a) bibliographic identification of the study; (b) objectives; (c) population; (d) methodological design*. In addition to these, other variables of interest were also included, such as: *(e) main findings on Dignity Therapy* and *(f) limitations*. To ensure the extraction sheet captured all the necessary information, a pilot test was conducted. For this, the same reviewers independently extracted the proposed data from five previously included articles. They then met to discuss the need for including additional variables. Following this discussion, the variable *(g) instruments used* was unanimously added to the final version of the extraction sheet, with approval from a third reviewer.

### Data analysis and presentation

The data were analyzed using the inductive qualitative content analysis technique proposed by Elo & Kyngäs [28], as described in the following procedures: in the first phase, *(a) data preparation*, the information extracted from the articles was organized into the extraction sheet according to the proposed variables. The reviewers then conducted multiple readings of the key findings to familiarize themselves with the dataset and identify an initial logical structure. In the second phase, *(b) open coding and development of a coding framework*, initial codes were created and grouped into emerging subcategories based on their semantic similarity. In the third phase, *(c) extraction and organization*, the subcategories identified in the previous phase were unified into broader textual categories based on content similarity. Finally, in the last phase, *(d) report preparation*, the final synthesis was integrated into the drafting of this review.

It is worth noting that the qualitative analysis was conducted exclusively by the lead author. Nonetheless, each stage was discussed in advance during team meetings, where decisions were debated, and procedures were defined.

Descriptive statistics (frequencies and percentages) were also applied to quantitatively summarize the numerical variables of the included studies. Finally, the qualitative results were presented through a narrative synthesis organized into textual categories, complemented by tables, charts, and graphs to facilitate the visualization and understanding of the findings.

## Results

In the initial search, 527 studies were identified, of which 51 peer-reviewed articles met the eligibility criteria and were included. Additionally, 355 documents were retrieved through the gray literature search; of these, only 7 peer-reviewed articles found via Google Scholar and 2 theses available on ProQuest met the established criteria and were added to the final sample. In total, 60 studies were included in this first stage.

**Fig. 1:**
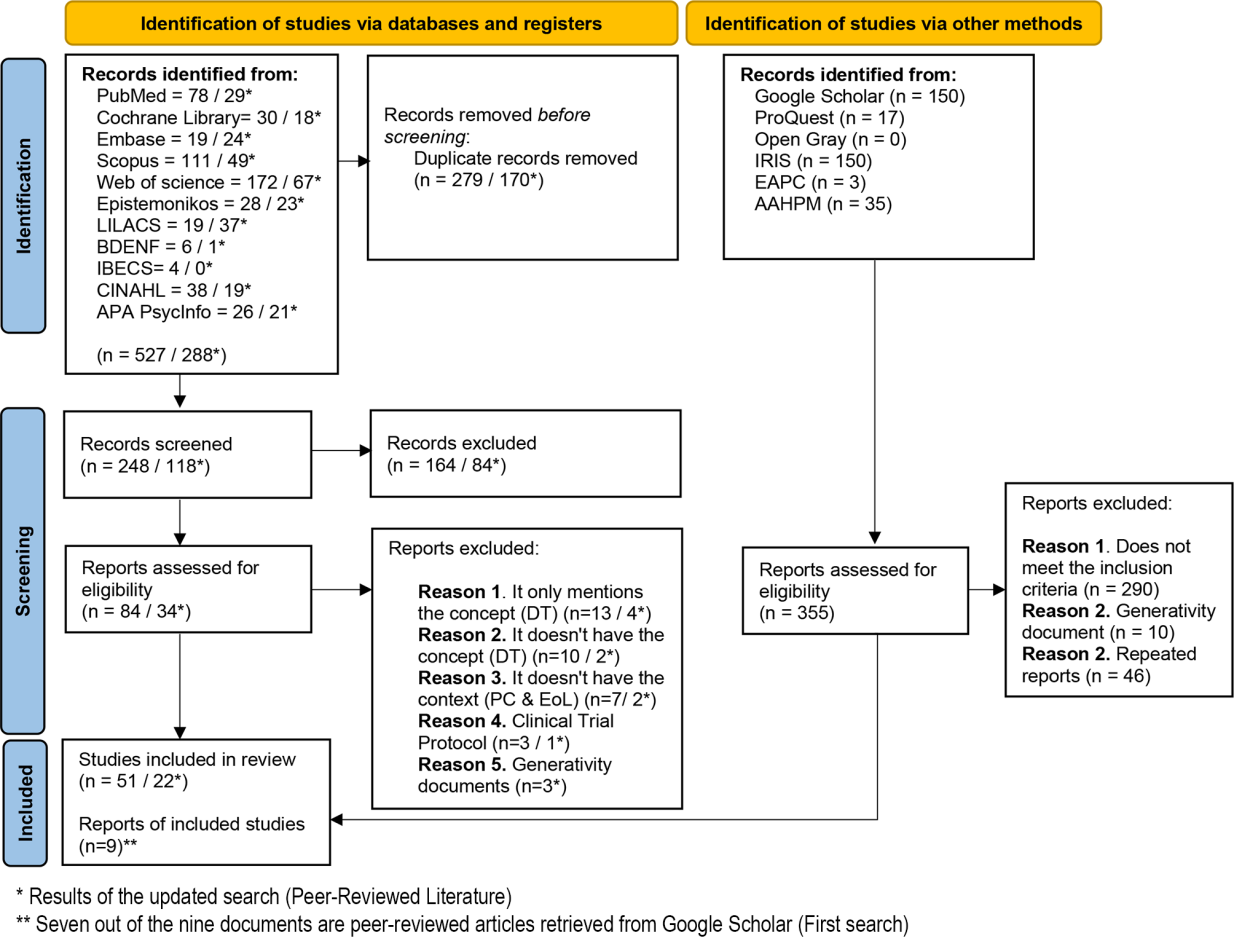
PRISMA Flowchart of the Literature Search and Selection Process. Flow diagram illustrating the identification, screening, and inclusion of studies in the scoping review. Adapted from Page et al. (2021)

In the updated search, 288 studies were identified, with 22 meeting the eligibility criteria and included in the review. Although the gray literature search was not updated, two relevant documents retrieved during this phase—although indexed in databases—were classified as gray literature and also included. As a result, the final sample consisted of 82 studies. The complete selection process is presented in Fig. 1.

### Description of the studies

Since 2016, most studies were concentrated in the period from 2023 to 2025 (*n* = 35; 42.6%), with 2023 being the year with the highest number of publications (*n* = 16; 19.5%). Regarding country of origin, most studies were conducted in the United States (*n* = 19; 23.1%), followed by China (*n* = 11; 13.4%) and Portugal (*n* = 7; 8.5%). In Latin America, only Brazil (*n* = 6; 7.3%) and Mexico (*n* = 2; 3.3%) have published studies on the topic.

With regard to methodological design, most studies were randomized controlled trials (*n* = 24; 29.2%), followed by review studies (*n* = 21; 25.6%) and mixed-methods studies (*n* = 8; 9.7%). A smaller proportion included quasi-experimental studies (*n* = 7; 8.5%), pilot studies (*n* = 5; 6%), and both methodological and qualitative studies (*n* = 4 each; 4.8%). In addition, letters to the editor, case reports, and case series were each represented by 3 studies (3.6%). Lastly, 8.5% (*n* = 7) consisted of abstracts presented at scientific events. The journals with the highest number of publications on the topic were *Palliative & Supportive Care* (*n* = 12; 14.6%), followed by the *Journal of Palliative Medicine* and the *Journal of Pain and Symptom Management* (*n* = 5 each; 6%).

Among the primary studies that included patients and applied Dignity Therapy (*n* = 53; 64.6%), most focused on oncological diseases (*n* = 31; 58.5%). However, its use was also notable in other chronic illnesses (*n* = 10; 18.9%) and in studies that included both groups of patients (*n* = 12; 22.6%).

With respect to the timing of therapy delivery, most studies implemented Dignity Therapy in advanced stages of illness (*n* = 44; 81.5%), while five studies (9.3%) applied it during early stages, and another five (9.3%) included patients at both stages of disease progression.

In terms of the individuals included in the studies, most were adults (*n* = 46; 85.2%). A smaller proportion documented the application of Dignity Therapy with children (*n* = 3; 5.6%) and adolescents (*n* = 2; 3.7%). In addition, several studies involved patients’ family members (*n* = 13; 24.1%) and healthcare professionals (*n* = 7; 13.0%).

The most commonly used assessment instruments were the *Patient Dignity Inventory* (*n* = 15; 28.3%) and the *Functional Assessment of Chronic Illness Therapy–Spiritual Well-Being Scale* (FACIT-Sp) (*n* = 8; 15%). Finally, the most frequently reported limitation across studies was the small number of participants included (*n* = 19; 35.8%). Additional characteristics of the included studies are presented in Table 1 and Supplementary Material 3.

**Table 1:** General characteristics of the included studies

Title	Authors	Year	Country	Journal	Study design	Aim	Population	Code**
“It seemed I was having a conversation with him”: Posthumous Dignity Therapy case series.	Julião M, Simões C, Chochinov HM	2025	Portugal	Palliative & Supportive Care	Case series	-	3 cases	01
The Qualitative DIGNISPACE Study: The Co-Design of a Life Review, Meaning-Making and Legacy Leaving Digital Intervention for Young People with Life-Limiting Conditions	Alison M. Rodriguez, et al.	2025	United Kingdom	Illness, Crisis & Loss	Qualitative	To co-design a digital Dignity Therapy-based intervention for young people with life-limiting conditions (DIGNISPACE)	Focus groups (*n* = 5) healthcare professionals (*n* = 23) young people with life-limiting conditions (*n* = 13) and family carers (*n* = 12).	02
Culturally Appropriate AI-Assisted Personalized Legacy Program for Patients With Serious Illness	Okere, S., Johnson, K.B, et al.	2025	USA	Journal of Pain and Symptom Management	Pilot study *	Adapting Artificial Intelligence-assisted legacy work program to address the unique needs of underrepresented minority patients with serious illnesses.	-	03
Efficacy of spiritual interventions in palliative care: An umbrella review of systematic reviews.	Austin, Philip D, et al.	2025	Australia	Palliative Medicine	Review	To systematically synthesise the available evidence from systematic reviews concerning (a) the efficacy of spiritual care interventions and (b) the extent and nature of spiritual care interventions used in specialist palliative care settings.	-	04
Dignity therapy led by a chaplain for palliative care patients in Brazil: an empirical study	Andressa Brant de Carvalho, et al.	2025	Brazil	Mortality	Qualitative	This study aimed to analyse the application effects of Dignity Therapy, led by a chaplain, with these patients.	10 patients	05
Dignity During a Pandemic: Dignity Therapy Delivered Through Telehealth Is Not Feasible in the Deep South	Reel, Candice D	2024	USA	The University of Alabama	Pilot study	The current study examined feasibility and efficacy of a telehealth delivery of the DT protocol to community dwelling hospice patients and their care partners and investigated challenges associated with hospice research recruitment through semi-structured interviews with hospice staff.	-	06
Study of Poetic Dignity Therapy for Sexual and Gender Minority Patients at End of Life	Memorial Sloan Kettering Cancer Center	2024	USA	ClinicalTrials.gov	RCT Protocol	The purpose of this study is to find out if dignity therapy is practical and works well for sexual and gender minority (SGM) patients in MSK	-	07
Development of the pediatric family-based dignity therapy protocol for terminally ill children (ages 7–18) and their families: A mixed-methods study	Junyi Lin, et al.	2024	China	Palliative & Supportive Care	Mixed methods	This study aims to develop a pediatric family-based dignity therapy (P-FBDT) protocol for terminally ill children and their families.	-	08
Telehealth dignity therapy for community-dwelling older adults: feasibility and potential efficacy	John Fallon; Sunil Bhar, et al.	2024	Australia	The Journal of Positive Psychology	Pilot study	We examined the feasibility and potential efficacy of Telehealth dignity therapy (TDT)	20 participants	09
The effect of dignity therapy on anxiety and depression in patients with chronic obstructive pulmonary disease: A randomized clinical trial	Sharifmoradi, T, Yousefi, H, et al.	2024	Iran	Journal of Education and Health Promotion	RCT	To investigate the effect of dignity therapy on the severity of anxiety and depression in patients with chronic obstructive pulmonary disease.	62 patients with chronic obstructive pulmonary disease	10
Translation and cross-cultural adaptation of the Posthumous Dignity Therapy Schedule of Questions to Brazilian Portuguese	Bennemann, Ana Carolina Kotinda, et al.	2024	Brazil	Palliative & Supportive Care	Methodological study	To translate the SQ for PDT into Brazilian Portuguese and adapt its content to suit the Brazilian Portuguese population	-	11
Effects of Dignity Therapy for Palliative Care Patients and Family Caregivers: A Systematic Review.	Haneef, Sara H, Abdullah, Marwah, et al.	2024	Saudi Arabia	Cureus	Review	This study aimed to systematically review the effects of DT on palliative care patients and their family caregivers, focusing on outcomes related to QoL, psychological distress (depression and anxiety), and overall well-being	-	12
Feasibility and acceptability of virtual dignity therapy for palliative care patients with advanced cancer	Wild, E., Weng, J., et al.	2024	USA	Journal of Pain and Symptom Management	Pilot study *	-	14 participants	13
Feasibility and Acceptability of Dignity Therapy for People with Advanced Neurodegenerative Disease	Labuschagne, D., Fleisher, J., Woo, K., Fitchett, G.	2024	USA	Journal of Pain and Symptom Management	Pilot study			14
Effects of dignity therapy on psychological distress and wellbeing of palliative care patients and family caregivers– a randomized controlled study	Seiler, A., Amann, M., et al.	2024	Switzerland	BMC Palliative Care	RCT	To evaluate whether DT can mitigate distress in both patients nearing the end of life and their family caregivers (FCs).	68 patients and their partners were randomly assigned to either DT, DT accompanied by their partner (DT +), or SPC.	15
Examining Moderation of Dignity Therapy Effects by Symptom Burden or Religious/Spiritual Struggles	Fitchett, G., Yao, Y., et al.	2024	USA	Journal of Pain and Symptom Management	RCT Secondary Analysis	To explore the effects of symptom burden and R/S struggles on DT outcomes.	579 participants with cancer, recruited from six sites	16
Advanced heart failure patients and family caregivers health and function: randomised controlled pilot trial of online dignity therapy	Yang, W., Zhang, X., et al.	2024	China	BMJ Supportive and Palliative Care	RCT	This research investigated the effectiveness of the caregiver mediated online dignity therapy in enhancing dyadic health and family function.		17
Effects of Dignity Therapy on individuals with amyotrophic lateral sclerosis: Case studies	Meira, M.D.V., Silva, R.S.D, et al.	2024	Brazil	Palliative & Supportive Care	Case series	To analyze the effects of Dignity Therapy (DT) on the physical, existential, and psychosocial symptoms of individuals with amyotrophic lateral sclerosis (ALS)	3 individuals with ALS	18
Development and Formative Evaluation of the Family-Based Dignity Therapy Protocol for Palliative Cancer Patients and Their Families: A Mixed-Methods Study	Chen, Z., Guo, Q. et al.	2024	China	Cancer Nursing	Mixed methods	The aims of this study were to develop a nurse-led psychotherapeutic intervention aiming to facilitate meaningful conversations between palliative cancer patients and their family members, named family-based dignity therapy (FBDT)	10 palliative cancer patients, 10 family members, and 13 oncology and hospice nurses	19
Cost considerations for implementing dignity therapy in palliative care: Insights and implications	Al Yacoub R; Rangel AP; et al.	2023	USA	Palliative & Supportive Care	Secondary Analysis of a randomized clinical trial	To examine the costs of implementing Dignity Therapy, including transcription, editing of the legacy document, and the time spent by dignity therapists on patient interviews and validation	317 patients randomly assigned	20
Translation and cross-cultural adaptation of the Dignity Therapy Question Protocol to Brazilian Portuguese.	Uchida Miwa M; Paiva CE; et al.	2023	Brazil	Palliative & Supportive Care	Methodological study	To translate and culturally adapt the Dignity Therapy Question Protocol (DTQP) to Brazilian Portuguese	41 participants	21
Effects of Dignity Therapy on Palliative Care Patients and their Partners. A Randomized Controlled Study	Seiler, A; Hertler, C; Schettle, M, et al.	2023	Switzerland	Palliative medicine	Randomized clinical trial*	To determine whether including a patient’s partner in Dignity Therapy (DT+) can mitigate distress in patients nearing the end of life and whether DT + can reduce grief-related distress in partners.	68 patients with a life expectancy of < 6 months and their partners	22
Chaplain and Nurse Implementation of Dignity Therapy: outcomes of Randomized Control Trial	Fitchett, G; Chochinov, H, et al.	2023	USA	Psycho-oncology	Randomized clinical trial*	To compare standard palliative care for outpatients with nurse-led and chaplain-led Dignity Therapy groups to determine their effects on patient outcomes and the impact on dignity	579 cancer patients recruited (age ≥ 55 years) who received outpatient palliative care	23
Dignity therapy effects by race: chaplain and nurse implementation in pragmatic, multisite steppedwedge randomized control trial	Wilkie DJ, Fitchett G, et al.	2023	USA	Cancer epidemiology biomarkers and prevention	Randomized clinical trial*	To compare standard palliative care for outpatients with nurse-led and chaplain-led Dignity Therapy groups to evaluate the primary effects on the impact of dignity and the interaction of Dignity Therapy with race.	579 cancer patients recruited (age ≥ 55 years) who received outpatient palliative care	24
Spiritual well-being, dignity-related distress and demoralisation at the end of life-effects of dignity therapy: a randomised controlled trial.	De Vincenzo F; Lombardo L; et al.	2023	Italy	BMJ Supportive & Palliative Care	Randomized clinical trial	To investigate the effectiveness of Dignity Therapy in improving spiritual well-being, reducing demoralization, and addressing dignity-related distress compared to standard palliative care	67 patients with terminal illnesses	25
The effect of Chinese culture-adapted dignity therapy on advanced cancer patients receiving chemotherapy in the day oncology unit: A quasi-experimental study.	Lin J; Guo Q; et al.	2023	China	European Journal of Oncology Nursing	Quasi-experimental study	To examine the effects of Dignity Therapy, adapted to Chinese culture, on dignity-related spiritual and psychological distress, as well as family functioning, in patients with advanced cancer undergoing chemotherapy in an outpatient oncology unit	39 patients	26
Effectiveness of dignity therapy in the context of culturally competent care in people with palliative care needs: a systematic review of systematic reviews.	Johnston B; Dönmez CF; Julião M;	2023	Portugal	Current Opinion in Supportive and Palliative Care	Overview	To synthesize evidence from systematic reviews and meta-analyses on the effectiveness of Dignity Therapy in relation to psychosocial and spiritual outcomes within the context of person-centered and culturally competent care	-	27
The European Portuguese Posthumous Dignity Therapy Schedule of Questions: Initial development and validation.	Julião M; Chochinov H; et al.	2023	Portugal	Palliative & Supportive Care	Methodological study	To develop, translate, and validate the Posthumous Dignity Therapy Question Schedule (p-DT-SQ) for administration to bereaved family members or friends	10 elderly individuals and 40 healthcare professionals	28
Dignity therapy, psycho-spiritual well-being and quality of life in the terminally ill: systematic review and meta-analysis	Zheng R; Guo Q; et al.	2023	China	BMJ Supportive & Palliative care	Review	To examine the effects of Dignity Therapy in randomized studies on various outcomes, including dignity, psycho-spiritual well-being, and quality of life, in terminally ill patients receiving palliative care but not active treatment	-	29
The Effect of Dignity Therapy on Terminally-Ill Adult Patients: A Systematic Review and Meta-Analysis	Lee, JL; Jeong, Y;	2023	South Korea	Iranian Journal of Public Health.	Review	To evaluate the effect of Dignity Therapy on dignity, distress, and quality of life in adult patients with terminal illnesses	-	30
Engaging Mortality: Effective Implementation of Dignity Therapy	Wilkie, D.J.; Fitchett, G.; et al.	2023	USA	Journal of Palliative Medicine	Randomized clinical trial	To compare standard palliative care for outpatients with Dignity Therapy led by a chaplain or a nurse to determine its effects on quality-of-life outcomes and the impact on dignity	579 patients	31
Implementing Dignity Therapy Service into an Acute Cancer Care Setting– A Feasibility Study	Claire Kelly, et al.	2023	Australia	Journal of Palliative Care	Randomized clinical trial	To evaluate the effectiveness and feasibility of introducing Dignity Therapy into a hospital cancer care service	15 patients	32
Evaluating the Impact of Dignity Therapy on Quality of Life in Patients with Brain Tumour: a Pilot Study	Mahiya Habib, Melissa B. Korman, et al.	2023	Canada	Journal of the Academy of Consultation-Liaison Psychiatry	Quasi-experimental study *	This study explored the impact of DT in patients with incurable BT earlier in the illness trajectory and at EOL.	39 of 43 participants completed DT across two groups: EOL (a prognosis of less than 1-year, *n* = 20), or non-EOL (a prognosis of 1-10years, *n* = 19)	33
Effectiveness of dignity therapy on well-being among patients under palliative care: A systematic review and meta-analysis	Bertha Wulandari, Erna Rochmawati	2023	Indonesia	International Journal of Nursing Studies	Review	To determine the effectiveness of Dignity Therapy in palliative patients, providing evidence that Dignity Therapy could be used in their care	-	34
A terapia da dignidade: uma intervenção especializada no conforto da pessoa em situação paliativa *[Dignity Therapy: a specialized intervention for the comfort of individuals in palliative care]*	Santos, Ana	2023	Brazil	Scientific Repository of the university	Scoping review	To map the effects of Dignity Therapy on patients in palliative care	-	35
Offering Dignity Therapy to a Muslim Patient–Caregiver Dyad Assisted in Palliative Care: Multidisciplinary Intervention with the Essential Role of an Official Interpreter	Buonaccorso, Loredana; et al.	2022	Italy	Journal of Palliative Medicine	Letter to the Editor	-	1 Muslim couple	36
Effects of family-oriented dignity therapy on dignity, depression and spiritual well-being of patients with lung cancer undergoing chemotherapy: A randomised controlled trial.	Xiao J; Chow KM; Choi KC, et al.	2022	China	International Journal of Nursing Studies	Randomized clinical trial	To examine the effectiveness of family-centered Dignity Therapy in improving dignity-related distress, depression, and spiritual well-being in Chinese patients with lung cancer undergoing chemotherapy	120 patients randomly assigned to receive family-centered Dignity Therapy	37
Development and feasibility of culturally sensitive family-oriented dignity therapy for Chinese patients with lung cancer undergoing chemotherapy.	Xiao J; et al.	2022	China	Asia-Pacific Journal of Oncology Nursing	Mixed methods	To develop and investigate the feasibility of a family-centered, evidence-based, and culturally sensitive Dignity Therapy intervention for Chinese patients with lung cancer undergoing chemotherapy	12 patient-caregiver dyads recruited	38
The effectiveness of dignity therapy on hope, quality of life, anxiety, and depression in cancer patients: A meta-analysis of randomized controlled trials	Zhang, YL; Li, JJ; Hu, XL; et al.	2022	China	International Journal of Nursing Studies	Review	To identify the effectiveness of Dignity Therapy on hope, quality of life, anxiety, and depression in patients with cancer	-	39
Dignity therapy for effective palliative care: a literature review	Se-Ryun Park; Yu-Jung Cha	2022	South Korea	Kosin Medical Journal	Review	To analyze the effectiveness and feasibility of Dignity Therapy in terminal patients through a review of previous studies	-	40
Dignity therapy in Mexican lung cancer patients with emotional distress: Impact on psychological symptoms and quality of life	Gonzalez-Ling, A; Vázquez, OG; et al.	2022	Mexico	Palliative & Supportive Care	Quasi-experimental study	To analyze the effect of Dignity Therapy on anxiety, depression, hopelessness, emotional distress, dignity-related distress, and quality of life in a group of Mexican patients with advanced lung cancer	29 patients	41
The Effectiveness of Online Dignity Therapy on Reducing Psychological Distress among Women with Metastatic Cancer	R Fallah, SA Mehrinezhad, et al.	2022	Iran	Quarterly Journal of Health Psychology	Quasi-experimental study	To examine the effectiveness of individual online Dignity Therapy in reducing psychological distress in women with metastatic cancer	30 women	42
Nursing, psychotherapy and advanced cancer: A scoping review.	Malakian Argin; Mohammed Shan, et al.	2022	Canada	European Journal of Oncology Nursing	Review	To map the literature on psychotherapeutic interventions among adults with advanced cancer and to explore the nursing role in this body of evidence.	-	43
Effects of dignity therapy on palliative patients’ family members: A systematic review.	Grijó L; Tojal C; Rego F, et al.	2021	Portugal	Palliative & Supportive Care	Systematic review	To explore the outcomes of Dignity Therapy for the family members of palliative care patients	-	44
Dignity Therapy for End-of-Life Care Patients: A Literature Review.	Cuevas PE; Davidson P; et al.	2021	USA	Journal of Patient Experience	Review	To explore the current state of empirical evidence supporting the use of Dignity Therapy in the care of patients facing end of life	-	45
Providing dignity therapy to patients with advanced cancer: a feasibility study within the setting of a hospital palliative care unit.	Francesca Nunziante; Silvia Tanzi	2021	Italy	BMC Palliative Care	Mixed methods	To assess the feasibility and acceptability of nurse-led DT intervention in advanced cancer patients receiving palliative care in a hospital setting in Italy.	A total of 37/50 patients were enrolled (74.0%), of whom 28 (75.7%) completed the assessment	46
Dignity Therapy Helps Terminally Ill Patients Maintain a Sense of Peace: Early Results of a Randomized Controlled Trial.	Iani L; De Vincenzo F; et al.	2020	Italy	Frontiers in Psychology	Randomized clinical trial	To investigate the effects of Dignity Therapy on specific dimensions of spiritual well-being, demoralization, and dignity-related distress in a sample of terminally ill patients	64 patients with terminal illnesses	47
Dignity therapy online: Piloting an online psychosocial intervention for people with terminal illness.	Bentley B; O’Connor M; et al.	2020	Australia	Digital Health	Quasi-experimental study	To examine the feasibility and acceptability of delivering Dignity Therapy through web-based support provided by a therapist to reduce costs, improve time efficiency, and promote access to treatment	6 participants	48
The Effectiveness of Dignity Therapy as Applied to End-of-Life Patients with Cancer in Taiwan: A Quasi-Experimental Study.	Li YC; Feng YH; et al.	2020	Taiwan	Asian Nursing Research	Quasi-experimental study	To determine the effectiveness of Dignity Therapy for cancer patients at the end of life	30 patients with cancer at the end of life, 16 in the experimental group and 14 in the control group	49
Dignity therapy for patients with brain tumours: qualitative reports from patients, caregivers and practitioners.	Korman MB; Ellis J; et al.	2020	Canada	Annals of Palliative Medicine	Qualitative study	To report on the feasibility of conducting Dignity Therapy with patients with brain tumors in their last year of life, as well as qualitative data on the acceptability and impact of Dignity Therapy collected from participating patients, their caregivers, and their Dignity Therapists	17 participants	50
Dignity Therapy in Pediatrics: A Case Series.	Schuelke T; Rubenstein J	2020	USA	Palliative Medicine Reports	Case series	To report the first case series of modified Dignity Therapy for a pediatric palliative care population	8 patients and their caregivers	51
Adapting the Portuguese dignity question framework for adolescents: ages 10–18.	Julião M; Antunes B; et al.	2020	Portugal	Palliative & Supportive Care	Methodological study	To adapt the Portuguese Dignity Therapy Question Framework for adolescents (DT-QF-Adol) aged 10 to 18 years	17 adolescents followed in an outpatient psychology clinic	52
The effect of dignity therapy on the quality of life of patients with cancer receiving palliative care	Zaki-Nejad, M.; et al.	2020	Iran	Iranian Journal of Nursing and Midwifery Research	Quasi-experimental study	To evaluate the effect of Dignity Therapy on the quality of life of a specific series of patients	50 cancer patients	53
Improving dignity of care in community-dwelling elderly patients with cognitive decline and their caregivers. The role of dignity therapy	Ounalli, H.; Mamo, D.; et al.	2020	Italy	Behavioral Sciences	Review	To provide a narrative review of current knowledge and recent evidence on Dignity Therapy in older adults with cognitive impairment	-	54
Randomized control trial of advanced cancer patients at a private hospital in Kenya and the impact of dignity therapy on quality of life	Weru, J; Gatehi, M; Musibi, A	2020	Kenya	BMC Palliative Care	Randomized clinical trial	To evaluate the effect of a single Dignity Therapy session on the quality of life of patients with advanced cancer	144 patients	55
Dignity Therapy in cancer patients: a systematized review of the literature	González-Ling, A; Galindo-Vázquez, O;	2020	Mexico	Gaceta Mexicana	Review	To analyze the existing literature on the effects of Dignity Therapy on anxiety, depression, dignity, and quality of life in patients with cancer	-	56
Dignity Therapy Improves Hope and Quality of Life in Cancer Patients: A Randomized Clinical Trial	Hossein Rahimi, et al.	2020	Iran	Journal of Advances in Medical and Biomedical Research	Randomized clinical trial	To determine the effect of Dignity Therapy on hope and quality of life in cancer patients	76 cancer patients	57
Stakeholder Perceptions of Dignity Therapy for Children and Young People with Life-Limiting and Life-Threatening Conditions in the UK	Lucy Watts, et al.	2020	UK	OBM Integrative and Complementary Medicine	Qualitative study	To explore the acceptability of Dignity Therapy for children and young people with life-limiting and life-threatening conditions, as well as for health professionals, and report the findings from stakeholder activities	80 healthcare professionals, 22 collaborators, 5 young individuals with life-limiting conditions.	58
To Honor and Bear Witness: A Clinician’s Reflection on Dignity Therapy for People Living with Dementia.	Aspiras DD; Empeño J; Montross-Thomas LP;	2019	USA	Journal of Palliative Medicine	Letter to the Editor	-	-	59
Application of dignity therapy in an advanced cancer patient — Wider therapeutic implications	Łabuś-Centek, M.; et al.	2019	Poland	Palliative Medicine in Practice	Case report	To describe the impact of Dignity Therapy on a patient with advanced cancer in a Polish hospital	1 patient	60
Effect of dignity therapy on quality of life in advanced cancer patients receiving palliative care atagakhan university hospital: a parallel group randomised control trial	Weru K, Gatehi M	2019	Kenya	Supportive care in cancer	Randomized clinical trial*	To evaluate the impact of Dignity Therapy (compared to standard care alone) on the quality of life of patients with advanced cancer receiving palliative care.	144 patients with advanced cancer (72 in each group)	61
Dignity Therapy as an aid to coping for COPD patients at their end-of-life stage.	Brożek B; Fopka-Kowalczyk M; et al.	2019	Poland	Advances in Respiratory Medicine	Randomized clinical trial	To assess overall feasibility and potential benefits of Dignity Therapy in patients with advanced Chronic Obstructive Pulmonary Disease	11 patients with severe Chronic Obstructive Pulmonary Disease	62
Dignity therapy and its impact on existential beliefs among generally healthy older adults: A mixed methods study.	Hughes, Desiree	2019	USA	Alliant International University	Mixed methods	To explore participants’ experiences with Dignity Therapy and whether it prompted reflection on existential themes	-	63
Effects of dignity therapy on dignity, psychological well-being, and quality of life among palliative care cancer patients: A systematic review and meta-analysis	Xiao, J.; Chow, K.M.; et al.	2019	China	Psycho-Oncology	Review	To identify the available evidence on the effects of Dignity Therapy on dignity, psychological well-being, and quality of life for cancer patients receiving palliative care	-	64
Effects and satisfaction of dignity therapy among patients with hematologic neoplasms in the Chinese cultural context: a randomized controlled trial	Chen, JY; Yan, J; et al.	2019	China	Supportive care in cancer	Randomized clinical trial	To evaluate the potential effects and satisfaction with Dignity Therapy among patients with hematologic malignancies in the Chinese cultural context	66 patients with hematologic malignancies	65
Effectiveness of dignity therapy for patients with advanced cancer: A systematic review and meta-analysis of 10 randomized controlled trials	Li, YF; Li, XX; et al.	2019	China	Depression and Anxiety	Review	To conduct a systematic review and meta-analysis to assess the overall effect of Dignity Therapy on anxiety, depression, dignity-related distress, and quality of life in patients with advanced cancer	-	66
Dignity therapy in palliative care: a bibliographic review	​​​​Molina Calle, Mariona	2019	Spain	Universidad de Girona	Review	To analyze the effectiveness of Dignity Therapy in improving outcomes for patients with potentially life-limiting advanced illnesses	-	67
The Effects of Dignity Therapy on Dignity at the End-of-Life	Amie Y. Bates	2019	USA	Pacific University Oregon	Review	To determine whether studies show an impact on dignity at the end of life through the use of Dignity Therapy	-	68
Effects of Dignity Therapy on Family Members: A Systematic Review.	Scarton LJ; Boyken L, et al.	2018	USA	Journal of Hospice and Palliative Nursing	Review	To provide a systematic review of the literature on the effects of Dignity Therapy on the family members of patients receiving this intervention	-	69
Dignity therapy interventions for young people in palliative care: a rapid structured evidence review.	Rodriguez A; Smith J; McDermid K	2018	UK	International Journal of Palliative Nursing	Rapid review	To summarise and synthesise the research that has explored dignity therapy and related meaning-making interventions in palliative care with young people.	-	70
Outcomes of a Dignity Therapy/Life Plan Intervention for Patients With Advanced Cancer Undergoing Chemotherapy.	Dose AM; McCabe PJ; et al.	2018	USA	Journal of Hospice and Palliative Nursing	Pilot randomized clinical trial	To examine the influence of combined interventions (Dignity Therapy plus Life Plan) on various psychosocial outcomes for individuals with advanced cancer receiving chemotherapy	18 patients with advanced pancreatic or lung cancer	71
Feasibility, acceptability and adaption of dignity therapy: a mixed methods study achieving 360° feedback.	Mai SS; Goebel S, et al.	2018	Germany	BMC Palliative Care	Mixed methods	To investigate the feasibility of Dignity Therapy in German Palliative Care Units (PCUs), as well as the acceptability and adaptation of a German version of the Dignity Therapy Question Protocol	30 participants	72
Development and evaluation of the Dignity Talk question framework for palliative patients and their families: A mixed-methods study.	Guo Q; Chochinov HM; et al.	2018	UK	Palliative Medicine	Mixed methods	To develop a novel tool called Dignity Talk to facilitate conversations, explore the anticipated benefits and challenges of using Dignity Talk, and gather suggestions for improving the protocol.	20 palliative care patients, 20 family members, and 34 healthcare providers	73
A narrative review of dignity therapy research.	Bentley, Brenda; O’Connor, Moira, et al.	2017	Australia	Australian Psychologist	Narrative review	To provide a narrative description of the literature on Dignity Therapy, offering a comprehensive review and critical synthesis of published research	-	74
The efficacy of dignity therapy on the psychological well-being in loved ones of terminally ill patients.	Julião, Miguel	2017	Portugal	Journal of Palliative Medicine	Letter to the Editor	To evaluate the effects of Dignity Therapy on the loved ones of terminally ill patients	25 participants, 15 randomized to Dignity Therapy and 10 to the Standard Palliative Care (SPC) group	75
Dignity Therapy and Life Review for Palliative Care Patients: A Randomized Controlled Trial.	Vuksanovic D; Green HJ, et al.	2017	Australia	Journal of Pain and Symptom Management	Randomized clinical trial	To compare Dignity Therapy with Life Review and a Waitlist Control group across a variety of outcome measures	70 participants	76
Feasibility and Acceptability of a Dignity Therapy/Life Plan Intervention for Patients With Advanced Cancer.	Dose AM; Hubbard JM; et al.	2017	USA	Oncology Nursing Forum	Pilot randomized clinical trial	To determine the feasibility and acceptability of a combined Dignity Therapy/Life Plan intervention in an outpatient oncology setting	18 patients within 12 months of diagnosis, undergoing treatment for advanced pancreatic cancer or lung cancer	77
Effect of dignity therapy on end-of-life psychological distress in terminally ill Portuguese patients: A randomized controlled trial.	Julião M; Oliveira F;	2017	Portugal	Palliative & Supportive care	Randomized clinical trial	To determine the influence of Dignity Therapy on demoralization syndrome (DS), desire for death (DfD), and sense of dignity (SoD) in terminally ill patients experiencing high levels of distress	80 participants	78
Integrating Dignity Therapy and Family Therapy in Palliative Care: A Case Study of Multiple Sclerosis, Depression, and Comorbid Cancer.	Ramos K; Fulton JJ;	2017	USA	Journal of Palliative Medicine	Case report	-	2 participants	79
Dignity therapy for adults with cancer receiving palliative care: A case report	Espíndola, A.V.; et al.	2017	Brazil	Temas em Psicologia	Case report	To evaluate the effectiveness of Dignity Therapy	1 patient	80
Comparing counseling and dignity therapies in home care patients: A pilot study.	Rudilla D; Galiana L, et al.	2016	Spain	Palliative & Supportive Care	Randomized clinical trial	To examine the effects of Dignity Therapy and counseling to provide actionable insights that could better address patients’ needs	70 patients assigned to two therapy groups	81
Living well with dementia: enhancing dignity and quality of life, using a novel intervention, Dignity Therapy	Bridget Johnston, et al.	2016	UK	International Journal of Older People Nursing	Mixed methods	To assess the feasibility, acceptability, and potential effectiveness of Dignity Therapy in improving quality of life and reducing psychological and spiritual distress in older adults with early-stage dementia	27 participants, including 7 individuals with ESD, 7 family members, 7 stakeholders, and 6 focus group members	82

* Conference abstracts

**Codes created to expand the data extracted from the included studies. Due to space constraints, additional variables for these same studies are presented in Supplementary Material 3

**Fig. 2:**
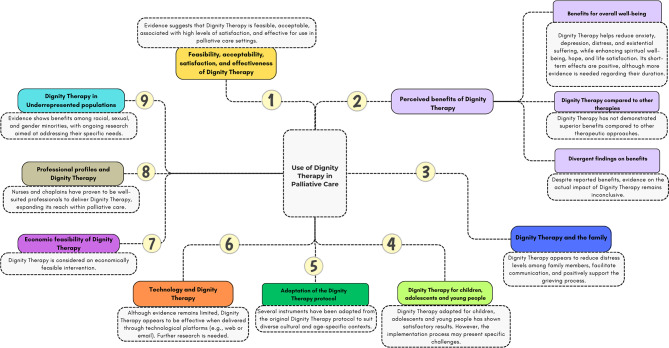
Inductive Qualitative Content Analysis: Definition of Categories and Subcategories. Diagram illustrating the identified categories and their corresponding subcategories derived from the inductive qualitative content analysis

From the basic qualitative content analysis, nine categories and three textual subcategories were identified. These are: feasibility, acceptability, satisfaction, and effectiveness; perceived benefits; Dignity Therapy and the family; Dignity Therapy for children, adolescents and young people; adaptation of the protocol; technology and Dignity Therapy; economic feasibility; professional profiles involved in delivering the intervention; and the implementation of Dignity Therapy in underrepresented populations. The categories and subcategories are detailed in Fig. 2 and discussed below.

### Category 1. Feasibility, acceptability, satisfaction, and effectiveness of dignity therapy

Although Dignity Therapy was developed in the early 2000s, interest in its implementation has grown significantly in recent years, alongside the advancement of palliative care. Several studies [29–39] have shown that Dignity Therapy is generally effective, feasible, acceptable, and well-received by both patients with advanced illnesses [31, 34, 35] and their family members [34, 37], who have reported high levels of satisfaction following its application [35, 37].

Dignity Therapy has also proven to be applicable and potentially effective in specific clinical contexts, such as individuals with early-stage dementia [29, 33, 40], advanced neurodegenerative diseases [39], and cancer during active treatment [32, 36]. Only one study reported limitations in its feasibility, related to emotional and organizational challenges encountered during implementation [38].

### Category 2. Perceived benefits of using dignity therapy

#### Subcategory 2.1. Benefits for overall well-being

In addition to being feasible and well accepted, Dignity Therapy has demonstrated meaningful benefits for patients’ overall well-being. Several studies have reported improvements across various health-related measures [13, 32, 35, 37, 38, 41–57], including physical functioning [56, 58, 59], quality of life [38, 56–58], sense of dignity [38, 45, 46, 55, 60], and social and family well-being [59, 61].

Benefits have also been observed in reducing distress related to physical symptoms [13], such as nausea, vomiting, insomnia, loss of appetite, and constipation [56], along with improvements in psychological distress [44, 46, 48]. In this sense, studies report reductions in levels of anxiety [62], depression, sadness [13, 38, 46, 58], hopelessness [46], desire for death, demoralization syndrome [48], and death anxiety [61].

In the existential dimension, improvements have also been reported in life satisfaction and self-esteem [47], as well as in the development of a more integrated personal narrative [38, 47, 63] and a greater sense of self-continuity [63]. These improvements have also been linked to the relief of suffering at the end of life [53, 54], particularly in patients with terminal or life-limiting illnesses [43, 51], and in those presenting with initial levels of emotional distress [64].

Aligned with its holistic approach, several studies have shown that Dignity Therapy has positive effects on spiritual well-being [37, 38, 41, 43, 51, 53, 54, 57, 64–66]. These benefits include an improved ability to recognize and address spiritual needs during the final stage of life [41], as well as relief from emotional distress related to faith [42]. Moreover, evidence suggests that Dignity Therapy maintains a positive impact on perceived dignity even among patients experiencing spiritual struggles or a high symptom burden, reinforcing its potential to support spiritual well-being in diverse settings [67].

Finally, the evidence also indicates that the effects of Dignity Therapy may persist beyond the immediate moment of its implementation, according to the evaluation time points defined in experimental designs. These benefits have been reported to last up to 7 and 14 days after the intervention [37, 52, 61], and in some cases, up to four weeks later [15, 49, 62, 68].

#### Subcategory 2.2. Benefits compared to other therapies

Given the existence of other psychotherapies with similar approaches, some studies have compared the benefits of Dignity Therapy to those of other interventions, assessing their impact on various dimensions of well-being. In the study by Vuksanovic et al. [35], Dignity Therapy showed better outcomes in terms of generativity and self-integrity compared to life review and a control group. However, no differences were found between groups regarding dignity-related distress or perceptions of quality of life.

Similarly, Rudilla et al. [69] observed that both Dignity Therapy and *Counseling* improved dignity, anxiety, spirituality, and quality of life. However, Dignity Therapy was associated with an increase in depression levels, while Counseling showed improvements in resilience and more marked reductions in anxiety.

Lastly, the study by Dose et al. [70], which compared Dignity Therapy with Life Plan Therapy, reported no significant differences in quality of life, spirituality, dignity, or sense of purpose, except for a reduction in distress observed at the three-month follow-up.

#### Subcategory 2.3. Divergent findings on benefits

Despite the broadly favorable evidence on the effects of Dignity Therapy, some studies have reported inconsistent results regarding its benefits, documenting either partial effects or no significant differences between intervention and control groups.

These studies show that, although Dignity Therapy appears to be effective, its impact on several health measures has been limited in some cases—particularly with regard to existential distress and sense of life meaning [14, 71], levels of anxiety or depression [62, 72, 73], and quality of life [18, 19, 68, 74]. Additionally, other studies have not identified statistically significant differences in the primary and secondary outcomes assessed [16, 38, 75], and some have even reported isolated adverse effects—such as an increase in anxiety levels in a single case [9].

Finally, Bentley et al. [76] point out that although Dignity Therapy is generally well accepted, it is not always effective, therapeutically valid, or practical, and may lead to family or cultural tensions.

### Category 3. Dignity therapy and the family

Although Dignity Therapy was originally conceived as a patient-centered intervention, several studies have explored its effects within the relational context, including the perceived impact on families, significant others, or caregivers in palliative care settings.

Some studies highlight that the family-adapted version of Dignity Therapy has been effective in improving quality of life, the Family APGAR Index—a measure of family functioning— [67, 77, 78], and spiritual well-being [79], as well as in reducing existential and psychological distress, including decreases in anxiety, depression, and emotional burden [31, 67, 77–79].

Several studies have also shown that Dignity Therapy can facilitate communication, recover positive memories, and strengthen family bonds [9, 11, 50, 79]. In addition, it has been found to promote reciprocity in the caregiving relationship, alleviate caregiver burden, and increase life satisfaction [12].

However, the study by Julião [10] did not identify statistically significant differences—either within the intervention and control groups or between them—in psychological well-being scores. Additionally, a systematic review highlighted the need for further research with adequate statistical power to conclusively evaluate the effects of Dignity Therapy on family members [17].

Finally, other studies have noted that the Legacy Document—produced through the therapy—can help patients and their loved ones prepare for the end of life and serve as a source of comfort and emotional support [53, 80].

### Category 4. Dignity therapy in children, adolescents, and young people

Although Dignity Therapy was originally designed for adults, in recent years its adaptation for children, adolescents, and young people has been explored in several studies, expanding its scope of application and opening new possibilities across different age groups [81–83].

These studies have shown that the intervention can facilitate open conversations about topics such as life, purpose, meaning, and death among children and adolescents, their families, and healthcare professionals [82, 83]. In particular, Watts et al. [83] observed that by evoking memories and encouraging reflection on what they consider important and how they wish to be remembered, Dignity Therapy can contribute to the psychosocial and spiritual well-being of young people with life-limiting or potentially life-threatening illnesses.

Complementing these findings, Rodriguez et al. [81] reported that young participants responded positively to the intervention and experienced statistically significant improvements in psychosocial well-being, emotional functioning, sense of dignity, and hope. The study also highlighted perceived benefits among family members.

### Category 5. Adaptation of the dignity therapy question protocol

The growing interest in Dignity Therapy has driven the adaptation of various instruments derived from the original question protocol developed by Chochinov, with the aim of applying it across different geographic, clinical, and age-related contexts [34, 84–86].

Among the adaptations identified in the literature, Miwa et al. [86] carried out a translation and cross-cultural adaptation of the protocol into Brazilian Portuguese, achieving a content validity index of 1 across all evaluated equivalences. Meanwhile, Mai et al. [34] translated and adapted the instrument into German for use in palliative care units, enabling its integration into local clinical contexts.

In Portugal, Julião et al. [85] developed a version of the protocol for posthumous use with bereaved family members. This version was later implemented, and the results indicated a predominantly positive response from participants, suggesting that it may serve as a valuable tool in bereavement support [87]. Building on this proposal, Bennenman et al. [88] adapted the posthumous Dignity Therapy protocol into Brazilian Portuguese to facilitate its application in local cultural contexts.

Additionally, two family-centered versions were developed in China. The first, aimed at children and adolescents with terminal illnesses, proposed a pediatric model based on meaningful interactions and creative activities, which was considered culturally appropriate by palliative care experts [89]. The second, focused on oncology patients and their families, was designed by nurses and inspired by elements of Chinese tradition. It was considered a promising intervention for facilitating end-of-life conversations [90].

In Canada, *Dignity Talk* was developed as a self-administered adaptation of the Dignity Therapy question protocol, designed to facilitate meaningful conversations between palliative care patients and their family members. The instrument was validated through a mixed-methods design involving patients, families, and professionals, who considered it accessible, clear, flexible, and emotionally respectful. Reported benefits include improved communication, strengthened family bonds, and the opportunity to address unfinished business [91].

Lastly, in Brazil, a version of the protocol was developed for adolescents aged 10 to 18 (DT-QF-Adol), with the aim of addressing the specific characteristics of this developmental stage and promoting communication tailored to their needs [84].

### Category 6. Technology and dignity therapy

In recent years, the integration of digital technologies has enabled the adaptation of Dignity Therapy to virtual environments, expanding its accessibility and range of application. In this context, several studies have reported that this modality is both feasible and acceptable [30, 92–94] and is associated with improvements in sense of meaning and life satisfaction [92], as well as reductions in psychological suffering [44].

Despite these positive results, some limitations have also been documented. For example, Reel [95] was unable to achieve patient adherence to the virtual modality, which prevented the evaluation of Dignity Therapy’s effectiveness in an online format. The author noted that this approach is not feasible in deep southern regions due to limited telehealth infrastructure and a lack of awareness about the value of the intervention. Similarly, Bentley et al. [30] pointed out that technological issues can pose challenges during implementation.

Regarding the creation of Legacy Documents in virtual format, Wild et al. [94] reported that for patients with greater physical decline, Dignity Therapy can require considerable effort, making it difficult to complete the document. Additionally, transcribing and editing the texts is a time- and resource-intensive process for interviewers [93, 94]. To improve the efficiency of this task, Wild et al.‘s team [94] developed a prototype of an artificial intelligence–assisted system aimed at optimizing the creation of Legacy Documents without compromising the human and cultural quality of the narratives.

Within this same context of digital innovation, *DIGNISPACE* was developed as a co-designed intervention based on Dignity Therapy, aimed at young people with life-limiting illnesses. Through a participatory process involving focus groups with professionals and interviews with young people and family caregivers, adaptations to the question protocol were identified, and a flexible, accessible digital application was designed. *DIGNISPACE* seeks to facilitate life review, construction of meaning, and legacy creation through digital tools tailored to the preferences and needs of this population [96].

### Category 7. Economic feasibility of dignity therapy

The economic feasibility of Dignity Therapy has been evaluated in several studies that examined the costs associated with its implementation and opportunities for optimization across different settings. The findings suggest that it is a relatively low-cost intervention, with the main expenses related to therapist compensation [49] and the transcription process of the Legacy Document [97].

Regarding the latter, Yacoub et al. [97] reported that the estimated total cost per protocol ranges between USD 331 and USD 356, including an average of USD 84.30 for the transcription and preparation of the Legacy Document, with variations depending on the type of therapist and care setting. Nevertheless, the authors emphasize that these costs are outweighed by the benefits of providing spiritual support at the end of life. In addition, Bentley et al. [30] highlighted that delivering Dignity Therapy in a virtual format can significantly reduce costs, further supporting its economic feasibility across different settings.

### Category 8. Professional profiles and dignity therapy

The expansion of professional profiles involved in delivering Dignity Therapy reflects a growing understanding that the quality of this intervention does not depend solely on the clinician’s specialty, but rather on their specific training, human sensitivity, and communication skills to support patients at the end of life.

In this sense, several studies have explored the role of nurses in delivering Dignity Therapy, highlighting them as well-suited therapists due to their comprehensive training and direct caregiving role with patients [38, 45, 55, 60, 65, 90, 98]. More recent research has also examined the role of chaplains, positioning them as effective facilitators of Dignity Therapy, particularly in settings where the spiritual dimension is a central component of the patient’s end-of-life experience [45, 60, 63].

### Category 9. Dignity therapy in underrepresented populations

The development of Dignity Therapy has led to its exploration in populations that have historically been underrepresented and often face greater inequities in access to these services, including both racial minorities and sexual and gender minorities. For example, the research by Wilkie et al. [45, 55, 60] showed that Dignity Therapy significantly improved the perception of dignity among cancer patients receiving outpatient palliative care, compared to standard care. This effect remained even after adjusting for sociodemographic variables such as age, sex, race, education, and income. Furthermore, although no statistically significant differences were observed in the intervention’s effect by race, the results showed a similarly positive impact among both white patients and those belonging to other racial minorities.

A protocol is also under development to evaluate the feasibility and effectiveness of Dignity Therapy among individuals who are part of sexual and gender minorities, including LGBTQ + populations. This study aims to adapt the intervention to the specific needs of these patients, promoting the expression of their life stories and the creation of a meaningful legacy that can be shared with their loved ones [99].

## Discussion

This review provides a comprehensive and up-to-date overview of Dignity Therapy in the context of palliative care, building upon previous studies [7, 18, 100]. The methodology employed extends beyond evaluating the therapy’s effectiveness, as it also explores its feasibility, acceptability, perceived health benefits, and implementation across various settings. These include its use in digital environments, its effects on pediatric and adolescent populations, and families, as well as associated costs, therapists’ profiles, and its use among underrepresented populations.

One notable trend in the retrieved literature is the increase in publications since 2016, with a peak in 2023. This growth may reflect the natural evolution of Dignity Therapy and its expanding role in palliative care. Factors such as the COVID-19 pandemic and population aging—associated with a higher prevalence of chronic illnesses—have likely contributed to the rising demand for palliative care, which in turn has driven further research in this field.

A geographic disparity was also observed among the included studies, with most publications originating from high-income countries (HICs), and only a small proportion from low- and middle-income countries (LMICs). This difference likely reflects long-standing inequalities in healthcare access. According to the Worldwide Hospice Palliative Care Alliance (WHPCA), approximately 76% of adults in LMICs have unmet palliative care needs [2]. In Latin America—where most countries fall within the LMIC category—the Atlas of Palliative Care [101] indicates that just 7% of individuals requiring such care actually receive it [102]. These figures help explain the limited number of studies from these regions.

A key finding of this review is the use of Dignity Therapy during the early stages of illness [10, 15, 33, 47, 60, 61, 92, 96], representing a significant expansion beyond the original methodology proposed by Chochinov [103]. Most studies continue to focus on patients with cancer, which is understandable given the high symptom burden associated with this disease [98, 102] and its status as a leading global cause of death [104]. However, the evidence also supports the use of Dignity Therapy in advanced non-oncological conditions [10, 12, 29, 30, 41, 47, 48, 59, 69, 71, 82], notably including patients with dementia [29, 33], who are often excluded from research due to cognitive impairment. This expanded application reinforces the potential of Dignity Therapy to address a wide range of psychosocial and spiritual needs in the context of palliative care, further demonstrating its versatility and relevance.

When it comes to health outcomes, findings remain inconclusive. Some studies report notable improvements in anxiety, depression, perceived dignity [13, 32, 35, 37, 38, 41–57], and spiritual well-being [37, 38, 41, 43, 51, 53, 54, 57, 64–66], while others found no statistically significant differences compared to standard palliative care [9, 14–16, 18, 19, 65, 68, 71–76] or other psychotherapies [35, 69]. Methodological variation across studies likely contributes to these inconsistencies, as does the qualitative nature of the intervention itself. The increasing number of global studies also reflects a diversity of cultural contexts, which may influence both how the therapy is implemented and how its effects are perceived. For instance, Martínez et al. [7] identified only five randomized controlled trials in their 2016 review, the first of which appeared in 2011 [105]. In contrast, the present review identified 24 experimental studies conducted since 2016, suggesting that some inconsistencies may stem from the field’s relatively recent development and ongoing evolution.

Bates [66] offers another explanation for these discrepancies, noting that in its early stages, Dignity Therapy lacked tangible outcomes, with benefits being largely subjective. As more specific and precise instruments were introduced to measure distress and other health-related dimensions, measurable effects became evident. While this is a plausible explanation, the introduction of such tools may have also contributed to variability in the findings. For example, “quality of life” is a frequently used construct—whether as a primary or secondary outcome—but a complex one, often evaluated without considering the short follow-up period—typically around 14 days—or the progressive clinical decline of patients in advanced illness. These factors can introduce bias in interpreting outcomes, particularly when improvement in quality of life is expected. This underscores the importance of carefully considering study design, the selection of constructs, and evaluation instruments. The timing of outcome measurements should also be taken into account in future research to better assess long-term effects.

The potential of Dignity Therapy to support family members is also well documented. Several studies have included relatives in post-intervention assessments [58, 77, 78] and adapted the original protocol for family settings [79, 89, 90]. These adaptations demonstrate the therapy’s capacity to reduce distress, improve spiritual well-being, and alleviate depressive symptoms among family members [67, 77, 78]. The intervention has also been shown to strengthen family communication and support preparation for the grieving process [9, 11, 50, 79]. These findings support the integration of Dignity Therapy into palliative care, acknowledging the patient–family unit as an interdependent relational system in need of coordinated care, as emphasized by Milberg et al. [106].

In pediatric and adolescent populations, Dignity Therapy also shows promise as a supportive intervention. However, implementation in this group presents unique challenges, related to developmental stages and communication dynamics, which typically involve healthcare providers, parents, and the child. In many cases, discussions about death are shaped by the parents’ understanding, personal experiences, and cultural beliefs. Adults often avoid addressing such topics with their children [107–109], adding complexity to the intervention’s application. Despite these challenges, Dignity Therapy has evolved through models that actively involve the family in the therapeutic process [89, 96], reflecting its capacity to adapt to the specific needs of different populations.

This adaptability is also evident in its application across diverse linguistic, clinical, and cultural settings. The literature points to a growing number of linguistic adaptations of the interview protocol designed to reflect local particularities [37, 75, 84–86, 88]. Posthumous uses of the therapy further illustrate its expansion into new clinical domains [85, 87, 88]. However, cultural expressions and religious frameworks remain underexplored and sparsely described, despite their potential influence on how patients experience dying and respond to the intervention. Cultural variability not only shapes acceptance but also perceptions of risks and benefits, ultimately affecting the therapy’s implementation and interpretation of outcomes. Dignity Therapy should therefore be understood and developed as a culturally competent intervention, with cultural dimensions integrated into both its design and delivery to align with the patient’s worldview [65].

Digital delivery represents another significant development in the trajectory of Dignity Therapy, especially in the post-pandemic era [110]. Studies show that digital formats—such as email, video conferencing, or mobile applications—can produce health outcomes comparable to those of in-person sessions [30, 44, 92–94]. This suggests considerable potential for extending the therapy’s reach and facilitating its use in varied settings. However, digital delivery may not be suitable in contexts with limited technological access or low levels of digital literacy [95], which poses additional challenges for its application. Disparities in access and technological skills can also affect perceptions of the therapy’s effectiveness, benefits, and risks, influencing both its acceptance and evaluation across different contexts.

Although limited, some evidence supports the cost-effectiveness of Dignity Therapy [49, 97], reinforcing its potential for implementation in low-resource settings. This is particularly relevant for LMICs, where access to specialized palliative care remains limited.

In line with this, the flexibility of the therapy protocol has enabled the involvement of a wide range of professionals. While Dignity Therapy was initially implemented by physicians or psychologists, more recent studies have included nurses—highlighting their suitability due to their training and direct caregiving relationship with patients [38, 45, 55, 60, 65, 90, 98]. There have also been positive reports of chaplains administering the intervention, which is notable given the therapy spiritual component [45, 60, 63]. Still, the presence of chaplains or similar professionals in palliative care settings remains limited or nonexistent, particularly in LMICs, as do training opportunities to adequately prepare them for this role.

Progress has also been made in applying Dignity Therapy among underrepresented populations in both research and practice. Studies have examined its impact on racial minorities [45, 55, 60], and initiated clinical trials focusing on sex and gender minorities [99], reflecting growing efforts to address the needs of these groups. These developments represent a significant step forward, but they also underscore the need for further research in these and other marginalized populations, including people experiencing homelessness, those in extreme poverty, individuals with disabilities, neurodivergent individuals, people affected by humanitarian crises, and those who are incarcerated—many of whom face greater unmet palliative care needs [2].

This review also identified several research gaps. Notably, there is a lack of studies evaluating Dignity Therapy in home care settings, despite their relevance in palliative care, particularly at the end of life. Additional research is needed to assess its effectiveness at different stages of illness, especially in conditions involving rapid functional decline.

Another gap concerns the limited reporting of potential confounding variables in experimental studies, such as concurrent access of patients and their families to spiritual, psychological, or social support services commonly included in palliative care. Although several studies account for these variables in their methodological design, the lack of transparency in reporting makes it difficult to accurately assess the specific contribution of Dignity Therapy to the observed outcomes.

The exclusion of individuals with linguistic difficulties or disabilities in existing studies is another concerning gap. Future research should explore how the therapy protocol can be adapted for these populations. Furthermore, given the qualitative nature of the intervention, future publications should provide detailed descriptions of the therapist’s role during the intervention. Such accounts should consider how therapists’ experiences, perspectives, values, and potential biases may influence both delivery and outcomes of the therapy. There is also a need to explore post-intervention effects on therapists themselves—an aspect currently absent from the literature.

Lastly, reflecting on the observations of Martínez et al. [7], future studies should prioritize the use of representative samples and ensure accurate calculations of sample size and statistical power. They should also clearly describe randomization procedures and provide details on who delivers the therapy and how it is conducted, as this information is often lacking. Strategies to reduce participant dropout and ensure adequate follow-up are also essential, given that many primary studies cite small sample size as a significant limitation.

This review has several notable strengths. It involved an extensive and recently updated search across eleven databases, included gray literature, and used descriptors in three languages—an approach that minimized the risk of missing relevant studies. In addition, the study’s design, implementation, and data analysis were carried out by a multidisciplinary team, which enriched interpretation through diverse perspectives. However, important limitations need to be considered. Despite the thorough search, some relevant studies may not have been identified—an inherent limitation of any review. Also, the inclusion of conference abstracts limited the amount of data available for analysis.

Finally, methodological heterogeneity among studies and variability in evaluated constructs made direct comparison of results difficult. The multidimensional nature of palliative care also meant that many studies contributed to more than one thematic category, occasionally leading to overlapping findings. To reduce this risk, we carefully reviewed each study’s content within its assigned category to ensure the selected data consistently reflected the core idea of each category.

## Conclusions

Dignity Therapy is a psychotherapy that has gained increasing interest and use in the field of palliative care. Available evidence suggests benefits across various dimensions of health particularly in the perception of dignity, relief of existential suffering, reduction of distress, anxiety, and depression, and improvements in spiritual well-being. However, some findings regarding its effectiveness remain inconclusive, which may be attributed to limitations in study designs and the qualitative nature of the intervention. Benefits have also been observed for family members, as well as for children, adolescents, and young people with life-limiting illnesses. The expansion of Dignity Therapy has led to the adaptation of its tools to different linguistic and geographic contexts, its application in various clinical settings, and the inclusion of a broader range of professionals in its delivery. As a brief and economically feasible intervention, further research is needed to address existing gaps, gain a deeper understanding of its impact, and support its integration into palliative care services.

### Practice implications

This review offers a broad overview of the evidence on the use of Dignity Therapy in palliative care, which may be useful for patients, healthcare professionals, and decision-makers interested in brief, low-cost interventions with the potential to enhance patient well-being throughout the course of illness. In addition, the findings of this review could guide discussions within palliative care teams and inform the design of future studies aimed at addressing the identified knowledge gaps. Nevertheless, the results should be interpreted with caution, as this review did not assess the risk of bias or the methodological quality of the included studies. Finally, it is recommended that the implementation of Dignity Therapy be carried out thoughtfully and with cultural sensitivity, given that in certain cases it may not produce the expected effects.

## Electronic supplementary material

Below is the link to the electronic supplementary material.

Supplementary Material 1

Supplementary Material 2

Supplementary Material 3

## Data Availability

No datasets were generated or analysed during the current study.
