# Supplementary material for: Use of dignity therapy in palliative care: a comprehensive scoping review
Source: BMC Palliat Care. 2025 Jul 1;24:177. doi: 10.1186/s12904-025-01812-4 (PMC12210638; doi:10.1186/s12904-025-01812-4)
Supplement: Supplementary file 2 — Supplementary Material 2 [file 12904_2025_1812_MOESM2_ESM.docx]

**Supplementary material 2.** Supplementary file of general search strategy.

| **Databases** | **Item searched** | **Filter** | **Retrieved (n)** | **Update** |
| --- | --- | --- | --- | --- |
| MEDLINE/PubMed | Concept  #1 (“Dignity therapy” [Title/Abstract] OR “Therapy of Dignity” [Title/Abstract:~0] OR “Dignity of Therapy” [Title/Abstract:~0])  Context  #2 (“Palliative care” [MeSH Terms] OR “Care, palliative” [Title/Abstract] OR “Palliative treatment” [Title/Abstract] OR “Palliative treatments” [Title/Abstract] OR “Treatment palliative” [Title/Abstract] OR “Treatments palliative” [Title/Abstract] OR “Therapy palliative” [Title/Abstract] OR “Palliative therapy” [Title/Abstract] OR “Palliative Medicine” [MeSH Terms] OR “Palliative care medicine” [Title/Abstract] OR “Palliative supportive care” [Title/Abstract] OR “Supportive care, palliative” [Title/Abstract] OR “Supportive care” OR “Terminal Care” [MeSH Terms] OR “Care, Terminal” [Title/Abstract] OR “End of Life Care” [Title/Abstract] OR “End-Of-Life Care” [Title/Abstract] OR “Care, End-Of-Life” [Title/Abstract] OR “End-Of-Life Cares” [Title/Abstract] OR “end of life” [Title/Abstract] OR “End-Of-Life” [Title/Abstract])  #3: #1 AND #2 | **Were applied manually within the strategy itself**  Mesh Terms [mesh terms] and Tittle/Abstract [tiab] | 78 | 29 |
| Cochrane Library | Concept  #1 ((“Dignity therapy” OR “Therapy of Dignity” OR “Dignity of Therapy”)):ti,ab,kw  Context  #2 ((“Palliative care” OR “Care, palliative” OR “Palliative treatment” OR “Palliative treatments” OR “Treatment palliative” OR “Treatments palliative” OR “Therapy palliative” OR “Palliative therapy” OR “Palliative Medicine” OR “Palliative care medicine” OR “Palliative supportive care” OR “Supportive care, palliative” OR “Supportive care” OR “Terminal Care” OR “Care, Terminal” OR “End of Life Care” OR “End-Of-Life Care” OR “Care, End-Of-Life” OR “End-Of-Life Cares” OR “end of life” OR “End-Of-Life”)):ti,ab,kw  #3: #1 AND #2 | **Were applied through the database**  Title Abstract Keywords  ( ):ti,ab,kw | 30 | 18 |
| Embase | Concept  #1 ('dignity therapy'/exp OR 'therapy of dignity':ti,ab OR 'dignity of therapy':ti,ab)  Context  #2 ('palliative therapy'/exp OR 'palliation':ti,ab OR 'palliative care':ti,ab OR 'palliative consultation':ti,ab OR 'palliative medicine':ti,ab OR 'palliative radiotherapy':ti,ab OR 'palliative surgery':ti,ab OR 'palliative therapy':ti,ab OR 'palliative treatment':ti,ab OR 'symptomatic treatment':ti,ab OR 'terminal care'/exp OR 'eol care':ti,ab OR 'end-of-life care':ti,ab OR 'terminal care':ti,ab OR 'end of life'/exp OR 'supportive care'/exp OR 'supportive therapy'/exp)  #3: #1 AND #2 | **Were applied through the database**  Title, Abstract or Author keywords*: ti,ab,kw* [for Emtree terms and synonyms]  Title or Abstract*: ti,ab* [for free terms] | 19 | 24 |
| Web of Science | Concept  #1 TS=((“Dignity therapy” OR “Therapy of Dignity” OR “Dignity of Therapy”))  Context  #2 TS=((“Palliative care” OR “Care, palliative” OR “Palliative treatment” OR “Palliative treatments” OR “Treatment palliative” OR “Treatments palliative” OR “Therapy palliative” OR “Palliative therapy” OR “Palliative Medicine” OR “Palliative care medicine” OR “Palliative supportive care” OR “Supportive care, palliative” OR “Supportive care” OR “Terminal Care” OR “Care, Terminal” OR “End of Life Care” OR “End-Of-Life Care” OR “Care, End-Of-Life” OR “End-Of-Life Cares” OR “end of life” OR “End-Of-Life”))  #3: #1 AND #2 | **Were applied through the database**  Topic includes title, abstract, keyword plus, and author keywords  Topic -TS=( ) | 172 | 67 |
| Scopus | Concept  #1 TITLE-ABS-KEY ((“Dignity therapy” OR “Therapy of Dignity” OR “Dignity of Therapy”))  Context  #2 TITLE-ABS-KEY ((“Palliative care” OR “Care, palliative” OR “Palliative treatment” OR “Palliative treatments” OR “Treatment palliative” OR “Treatments palliative” OR “Therapy palliative” OR “Palliative therapy” OR “Palliative Medicine” OR “Palliative care medicine” OR “Palliative supportive care” OR “Supportive care, palliative” OR “Supportive care” OR “Terminal Care” OR “Care, Terminal” OR “End of Life Care” OR “End-Of-Life Care” OR “Care, End-Of-Life” OR “End-Of-Life Cares” OR “end of life” OR “End-Of-Life”))  #3: #1 AND #2 | **Were applied through the database**  Doc Title, Abstract, Keyword  TITLE-ABS-KEY ( ) | 111 | 49 |
| Epistemonikos | Concept  #1 (title:(("Dignity therapy" OR "Therapy of Dignity" OR "Dignity of Therapy")) OR abstract:(("Dignity therapy" OR "Therapy of Dignity" OR "Dignity of Therapy")))  Context  #2 (title:(("Palliative care" OR "Care, palliative" OR "Palliative treatment" OR "Palliative treatments" OR "Treatment palliative" OR "Treatments palliative" OR "Therapy palliative" OR "Palliative therapy" OR "Palliative Medicine" OR "Palliative care medicine" OR "Palliative supportive care" OR "Supportive care, palliative" OR "Supportive care" OR "Terminal Care" OR "Care, Terminal" OR "End of Life Care" OR "End-Of-Life Care" OR "Care, End-Of-Life" OR "End-Of-Life Cares" OR "end of life" OR "End-Of-Life")) OR abstract:(("Palliative care" OR "Care, palliative" OR "Palliative treatment" OR "Palliative treatments" OR "Treatment palliative" OR "Treatments palliative" OR "Therapy palliative" OR "Palliative therapy" OR "Palliative Medicine" OR "Palliative care medicine" OR "Palliative supportive care" OR "Supportive care, palliative" OR "Supportive care" OR "Terminal Care" OR "Care, Terminal" OR "End of Life Care" OR "End-Of-Life Care" OR "Care, End-Of-Life" OR "End-Of-Life Cares" OR "end of life" OR "End-Of-Life")))  #3: #1 AND #2 | **Were applied through the database**  (title:(( )))  (abstract:(( ))) | 28 | 23 |
| APA PsycInfo | Concept  #1 Abstract: (“Dignity therapy” OR “Therapy of Dignity” OR “Dignity of Therapy”)  Context  #2 Abstract: (“Palliative care” OR “Care, palliative” OR “Palliative treatment” OR “Palliative treatments” OR “Treatment palliative” OR “Treatments palliative” OR “Therapy palliative” OR “Palliative therapy” OR “Palliative Medicine” OR “Palliative care medicine” OR “Palliative supportive care” OR “Supportive care, palliative” OR “Supportive care” OR “Terminal Care” OR “Care, Terminal” OR “End of Life Care” OR “End-Of-Life Care” OR “Care, End-Of-Life” OR “End-Of-Life Cares” OR “end of life” OR “End-Of-Life”)  #3: #1 AND #2 | **Were applied through the database**  Abstract | 26 | 21 |
| LILACS | Concept  #1 (“Dignity therapy” OR “Terapia de la dignidad” OR “Terapia da dignidade”)  Context  #2 (“Palliative care” OR “Cuidados paliativos” OR “Terminal care” OR “Cuidados de fin de vida” OR “Cuidados de fim de vida”)  #3: #1 AND #2 | **No filters applied** | 19 | 37 |
| BDENF |  |  | 6 | 1 |
| IBECS |  |  | 4 | 0 |
| CINAHL | Concept  #1 "Dignity therapy" OR "Therapy of Dignity" OR "Dignity of Therapy"  Context  #2 (MM "Palliative Care") OR (MM "Palliative Care Nursing") OR (MM "Palliative Medicine") OR (MM "Palliative Care Nurses") OR (MM "Terminal Care+") OR "supportive care" OR "End of Life Care"  #3: #1 AND #2 | **Were applied through the database**  AB  (Abstract or Author-Supplied Abstract) | 38 | 19 |
| **Organizations, Virtual Libraries and Websites and grey literature** | | | |  |
| Google Scholar | "Palliative care" OR "Terminal care" OR "End of life Care" AND "Dignity Therapy" | **No filters applied** | 150 | - |
| The ProQuest Dissertation & Theses Global | ("Palliative care" OR "Terminal care" OR "End of life Care") AND ("Dignity Therapy") |  | 17 | - |
| Open Gray | ("Palliative care" OR "Terminal care" OR "End of life Care") AND ("Dignity Therapy") |  | 0 | - |
| IRIS WHO | Palliative care OR Terminal care OR End of life Care AND Dignity Therapy |  | 150 | - |
| EAPC | Dignity therapy AND Palliative care |  | 3 | - |
| AAHPM | Dignity therapy AND Palliative care |  | 35 | - |
