# Supplementary material for: Use of dignity therapy in palliative care: a comprehensive scoping review
Source: BMC Palliat Care. 2025 Jul 1;24:177. doi: 10.1186/s12904-025-01812-4 (PMC12210638; doi:10.1186/s12904-025-01812-4)
Supplement: Supplementary file 3 — Supplementary Material 3 [file 12904_2025_1812_MOESM3_ESM.docx]

**Supplementary material 3**. General characteristics of the included studies. *Con*t.

| **Code** | **Key findings** | **Instruments** | **Limitations** |
| --- | --- | --- | --- |
| 01 | Their overwhelmingly positive response (p-DT) suggests this approach holds promise as a novel bereavement intervention.  Findings suggest p-DT may serve as a valuable tool for bereavement support, warranting further research to expand its scope and accessibility | Posthumous Dignity Therapy Protocol | - |
| 02 | DIGNISPACE addresses the psycho-spiritual needs of young people with life-limiting conditions.  DIGNISPACE is the first co-designed Dignity-Therapy-based intervention for young people that can be delivered digitally. It is proposed that DIGNISPACE will complement the excellent work already undertaken in hospices and palliative care teams by providing a supported, structured intervention for young people (and where appropriate families) to engage in life reflection, meaning making and legacy leaving. | - | - |
| 03 | During this trial we found that the editing and transcription process was time and resource intensive and consequently have prototyped an AI-assisted pipeline to enhance efficiency of the process. In addition, the included patient population primarily encompassed non-Hispanic White patients, consistent with the limited representation of minority patients in much of the research on legacy work. We will utilize focus groups within the community to assess barriers to participation in studies utilizing AI and address unique adaptations that should be made to the intervention for underrepresented minorities. | - | - |
| 04 | Findings show that spiritual care interventions especially dignity therapy and life-review may be effective for improving outcomes including spiritual wellbeing, emotional symptoms, quality-of-life and physical symptoms in people receiving specialist palliative care. Under half of included reviews report follow-up data where only emotional symptoms and quality-of-life are reported at more than one time-point. | - | - |
| 05 | The results suggest that DT fulfils its purpose, proving to be a valuable tool for chaplains to use in palliative care to promote patients’ dignity at the end of life. Professional chaplains trained in DT can significantly contribute to quality spiritual care for palliative care patients. | - | - |
| 06 | Results of feasibility showed three potential participants were recruited but none consented to participate. The results from the feasibility study precluded our ability to assess efficacy as planned. Seven members of the hospice staff completed qualitative interviews designed to understand the lack of feasibility of this study. Results identified four main themes that point to the value of the DT intervention, an overwhelming disapproval of telehealth delivery of interventions, a close consideration of research methods, and the need for future research to further the advancement and clinical use of this effective intervention, particularly in rural and underserved areas | - | - |
| 07 | An Open Trial of a Novel Poetic Dignity Therapy Intervention to Enhance Meaning and Purpose for Sexual and Gender Minority Patients With Cancer | - | - |
| 08 | The P-FBDT protocol was recognized as highly reasonable and the P-FBDT interview guide was endorsed as important, acceptable, clear, comprehensive, and suitable to be used in pediatric palliative care practice in Chinese culture (>90%). Potential benefits, possible challenges, and practical considerations of the P-FBDT were also proposed. | - | The protocol has not been evaluated from the perspectives  of terminal children and their families, which will be done  in future feasibility and pilot studies. Besides, participants in this study were predominantly female, which is representative for the pediatric palliative care workforce dominated by women in China; however, how male perspectives could inform the study findings needs to be addressed in future studies. |
| 09 | Participants’ MiL (Meaning in Life) and life satisfaction significantly improved between before and after TDT, and MiL gains were maintained at three-month follow-up. The program was found to be a feasible, timely, and potentially efficacious positive psychology intervention for community-dwelling older adults. TDT is feasible, acceptable and associated with improvements in MiL and life satisfaction for community dwelling older adults without life limiting illnesses | - Structured Mini-Mental State Examination - Participant Feedback Questionnaire - Multidimensional Existential Meaning Scale - Northwestern Ego-Integrity Scale - The Satisfaction with Life Scale - Positive and Negative Affect Schedule - Centre for Epidemiological Studies Depression Scale | There are several limitations to these findings. First, as the present study did not include a comparator group, findings regarding efficacy are preliminary. Second, our sample was not representative of the population. Namely, participants in this study were mostly female, highly educated, and born in Australia. Third, along with the lack of diversity in our sample, the small sample size further limits the generalisability of our findings. |
| 10 | The mean score of anxiety of the patients before the intervention was not significantly different between the two groups (*P* = 0.18); but one month after the intervention, it was significantly lower in the intervention group than in the control group (*P* = 0.05). Also, the score of depression was not significantly different between the two groups before (*P* = 0.68) and one month after the intervention (*P* > 0.05). Dignity therapy could reduce anxiety in patients with chronic obstructive pulmonary disease; thus, it could be used as a nonpharmacological, cost-effective and probably without side effects method. | Hospital Anxiety and Depression Scale | Among the limitations of the present study, mention may be made of the small sample size, the implementation of the intervention in only one treatment center, and the blinding of the samples |
| 11 | The questionnaire was translated, back-translated, and evaluated by the panel of experts, obtaining a Content Validity Index of 0.97. During the pretest phase, it was observed that the participant’s interview method needed to be changed from remote (telephone or videoconference) to in-person. Additionally, it was necessary to modify some terms related to death and dying, as they caused discomfort to the participants. As a result of this process, the PDT was modified, and adapted to the Brazilian cultural and linguistic reality. | The Posthumous Dignity Therapy Schedule of Questions (PDT-SQ) | The principal limitation of this study was that it was restricted to one center in Brazil in a city within the interior of Paraná state (Brazil). Another limitation is that while we have established the validity of the adapted schedule of questions, we have yet to implement and evaluate its impact within a sample of care providers. |
| 12 | Eight RCTs met the inclusion criteria. DT demonstrated significant improvements in QoL and well-being among palliative care patients, with reductions in depression and anxiety. Improvements were observed in the physical, psychological, and social domains of QoL. Family caregivers also experienced enhanced spiritual well-being, hope, family cohesion, and adaptability. However, not all outcomes were consistently improved across all studies, indicating variability in DT effectiveness. | - | Variability in the methodological quality of the included studies is a significant concern. Several studies exhibit high risk in domains such as blinding and allocation concealment, which can affect the robustness of the review's findings. |
| 13 | Fourteen patients consented. In completed program evaluation surveys, acceptability of the virtual component (4.6/5 rating) and satisfaction with the DT process (4.4/5 rating) were high (n=5). Post DT intervention interviews were also conducted. Results support to the feasibility and acceptability of vDT. For patients that are too sick DT can be high effort not allowing for completion of the GD. Transcribing and editing the document can be time intensive for the interviewer. Finding ways to move vDT into clinical practice is important. | - | - |
| 14 | We demonstrated that DT was feasible and well-received in the NPC setting. | - | - |
| 15 | The coalesced group (DT and DT +) revealed a significant increase in patients’ perceived quality of life (FACIT-Pal-14) following the intervention (mean difference 6.15, SD = 1.86, *p* < 0.01). We found a statistically significant group-by-time interaction effect: while the HADS_tot_ of patients in the intervention group remained stable over the pre-post period, the control group’s HADS_tot_ increased (F = 4.33, df = 1, 82.9; *p* < 0.05), indicating a protective effect of DT. Most patients and their FCs found DT useful and would recommend it to other individuals in their situation. | - The Hospital Anxiety and Depression Scale - The Distress Thermometer - Functional Assessment of Chronic Illness Therapy Palliative Care (FACIT-Pal) and Spiritual Wellbeing Scale (FACIT-SP) - Patient Dignity Inventory—German Version - WHO Quality of Life Questionnaire   PRISM (Pictorial Representation of Illness and Self Measure) | Firstly, the study was restricted by its small sample size. As a consequence, the anticipated number of patients calculated in the power analysis was not reached, potentially hindering the ability to detect subgroup effects. Second, the low participation and the high attrition rate poses an additional constraint on our study, thereby further limiting reliability and interpretation of our results. Third, it is important to note that floor effects for the primary outcome (HADS ≥ 8) could potentially exert a substantial influence on the outcome findings and the interpretation of the study results. |
| 16 | Neither baseline symptom burden nor R/S struggle significantly moderated the effect of DT on DIS in this sample. For patients, the difference over time was significant in QoL (p<0.001). The interaction effects were significant for hope (p<0.001), well-being (p<0.001), Family APGAR Index (p<0.001) and QoL (p=0.007). For family caregivers, a significant difference in depression (p=0.001) was found within groups. Meanwhile, the interaction effects were significant on anxiety (p=0.002) and depression (p=0.016). | - 7-Item Dignity Impact Scale (DIS) - QUAL-E subscales (preparation for death; life completion); - Edmonton Symptom Assessment Scale (ESAS-r)   Religious Spiritual Struggle Scale (RSS-14; R/S). | The present study was limited by our inability to recruit more participants with high symptom burden or R/S struggle; a problem faced by other palliative care investigators. Additionally, although we followed recommendations from other investigators, existing research points to diverse cutpoints for the ESAS symptoms and support for the cutpoint we selected for the RSS-14 is limited. |
| 17 | For patients, the difference over time was significant in QoL (p<0.001). The interaction effects were significant for hope (p<0.001), well-being (p<0.001), Family APGAR Index (p<0.001) and QoL (p=0.007). For family caregivers, a significant difference in depression (p=0.001) was found within groups. Meanwhile, the interaction effects were significant on anxiety (p=0.002) and depression (p=0.016). Caregiver-mediated online dignity therapy among patients with advanced HF had potential to enhance patient outcomes (level of hope, well-being, family function and QoL) and alleviate caregiver outcomes (anxiety, depression) at 4-week and 8-week follow-up. | - Herth hope index (HHI) - The General Well-Being Schedule (GWBS) - The Minnesota living with HF - Generalized Anxiety Disorder 7-item (GAD-7) for family caregivers - Patient Health Questionnaire-9 (PHQ-9) for family caregivers   The Family APGAR Index for patients and family caregivers | First, the pilot study involved a small sample size and was conducted only in Tianjin, a province in China. Second, the positive change in the treatment group may be due in part to the Hawthorne effect, since the participants in dignity therapy were not blinded and the primary outcome variable was assessed using a questionnaire with subjective patient responses, although the questionnaire has good confidence. Lastly, the follow-up time of this pilot study was short, and the long-term effects of the intervention are yet to be determined. |
| 18 | The scale results indicate that DT led to an improvement in the assessment of physical, social, emotional, spiritual, and existential symptoms according to the score results. It is worth noting that the patient with a recent diagnosis showed higher scores for anxiety and depression after DT. Regarding the PDI, the scores indicate improvements in the sense of dignity in all 3 cases, which aligns with the positive verbal reports after the implementation of DT. | - Patient Dignity Inventory - Hospital Anxiety and Depression Scale - Functional Assessment of Chronic Illness Therapy-Spiritual Well-Being   Edmonton Symptom Assessment Scale | This study has strengths and limitations that need to be acknowledged. The small sample size and study design do not allow for the generalization of findings. However, the observed results provide a basis for more robust studies to test the benefits of DT for patients with ALS and other chronic degenerative diseases. |
| 19 | The FBDT interview guide was endorsed by most palliative cancer patients and family members (>75.0%), as well as oncology and hospice nurses (>90.0%). Potential perceived benefits and challenges of FBDT were proposed by participants. The FBDT protocol was modified according to feedback from participants to make it more suitable to use in clinical practice in China.  The FBDT was perceived to be a potentially promising intervention to facilitate meaningful end-of-life conversations among palliative cancer patients and family members in China. | - | - |
| 20 | The benefits of providing spiritual support at the end of life with Dignity Therapy outweigh the costs.  Research findings show that Dignity Therapy is a relatively low-cost intervention. | - | A limitation of the study was the cost involved in delivering Dignity Therapy and editing the transcriptions into legacy documents. Additionally, there was a lack of precision regarding the amount of time required for these tasks |
| 21 | The Portuguese version of the DTQP – Dignity Therapy Question Protocol – demonstrated a content validity index of 1 for all equivalences. The final version consisted of 10 questions, which were approved by the original authors, who confirmed that the Brazilian Portuguese version of the DTQP retained the original characteristics of the English version | - | - |
| 22 | The study did not reveal significant differences before and after the intervention or between the groups regarding primary and secondary outcomes. However, a statistically significant interaction effect between group and time was found: while the HADStot of patients in the Dignity Therapy group remained stable during the pre-post period, the HADStot of the control group increased (F = 4.33, df = 1, 82.9; p = 0.044), indicating a protective effect of Dignity Therapy. Most patients and their partners found Dignity Therapy useful and would recommend it to others in similar situations. | - Hospital Anxiety and Depression Scale | - |
| 23 | In the pre-test, the mean DIS score was 24.3 ± 4.3 in the Dignity Therapy group and 25.9 ± 4.3 in the standard care group. After adjusting the pre-test DIS scores for study location and study phase, the chaplain-led (β = 1.7, p = 0.02) and nurse-led (β = 2.1, p = 0.005) groups reported significantly higher post-test DIS scores than the standard care group. After adjusting for age, gender, race, education, and income, the effect on DIS scores remained significant for both Dignity Therapy groups. Whether led by chaplains or nurses, Dignity Therapy was effective in improving the impact on the dignity of outpatient cancer patients receiving palliative care. | - Dignity Impact Scale | - |
| 24 | After examining the interaction between race and Dignity Therapy for the entire sample, we found that the interaction was not significant (p = 0.73), and the effect sizes of Dignity Therapy were similar for white patients (β = 1.9, p = 0.005) and for patients of other races (β = 1.6, p = 0.055) | - Dignity Impact Scale | - |
| 25 | The general linear model for repeated measures showed significant differences between groups in terms of peace and psychological distress over time, but not in existential distress, physical distress, meaning and purpose, distress and coping ability, or meaning and faith. Specifically, patients in the Dignity Therapy group maintained similar levels of peace from baseline through follow-up, while patients in the control group showed a significant decrease in peace during the same period. Additionally, psychological distress significantly decreased from pre-treatment to post-treatment in the intervention group and increased in the control group | - FACIT-Sp-12 - Demoralization Scale-II - Patient Dignity Inventory | The small sample size and high attrition rate, primarily due to patient deaths, may have limited the study's power when considering the final sample |
| 26 | There was no statistical significance in the results at T1 between the two groups, nor in most of the results between T0 and T1 within the intervention groups, except for the relief of dignity-related distress (P = 0.017), particularly physical distress (P = 0.026), and the improvement in family functioning (P = 0.005), especially family adaptability (P = 0.006). The synthesized quantitative and qualitative results showed that the intervention could alleviate physical and psychological distress, enhance the sense of dignity, and improve spiritual well-being and family functioning in patients | - Distress thermometer (DT) - FACIT-Sp-12 - Family Adaptability and Cohesion evaluation scale-II (FACES II) - Patient Dignity Inventory | The use of a pilot quasi-experimental design, which offers less rigor than a randomized clinical trial (RCT), limits the findings.  The study was conducted in a single oncology unit, restricting generalizability.  The small sample size impacts the robustness of the results, though they are partly supported by qualitative data.  Additionally, the lack of long-term effect evaluation, with assessments only at baseline and post-intervention, is a limitation |
| 27 | Dignity Therapy has a positive impact on anxiety, depression, suffering, and the meaning and purpose of life for individuals with palliative care needs. However, the evidence is somewhat contradictory regarding whether Dignity Therapy is effective in improving hope, quality of life, and spiritual outcomes in the context of culturally competent care. Nurse-led Dignity Therapy appears desirable given the critical role nurses play in caring for individuals with palliative care needs | - | - |
| 28 | The p-DT-SQ demonstrated significant content validity (CVC = 0.94) and apparent validity: it was considered clear, easy to understand, of reasonable length, and not difficult to answer. Participants felt comfortable responding to the p-DT-SQ and believed it could positively affect how they or others would remember their loved ones, allowing for a better understanding of the concerns, interests, and values of the deceased. | - | - |
| 29 | Dignity Therapy did not improve the sense of dignity (p = 0.90), hope (p = 0.15), spiritual well-being (p = 0.99), or quality of life (p = 0.23) in terminally ill patients. However, Dignity Therapy reduced anxiety and depression after the intervention (standardized mean difference, SMD = -1.13, 95% CI (-2.21 to -0.04), p = 0.04; SMD = -1.22, 95% CI (-2.25 to -0.18), p = 0.02, respectively), and 4 weeks after the intervention (SMD = -0.89, 95% CI (-1.71 to -0.07), p = 0.03; SMD = -1.26, 95% CI (-2.38 to -0.14), p = 0.03, respectively) | - | The effects of Dignity Therapy were not addressed in patients who were not in the final stage of life or receiving palliative care |
| 30 | Dignity Therapy may be effective for terminally ill patients in terms of dignity and anxiety | - | - |
| 31 | Chaplain-led or nurse-led Dignity Therapy was effective in improving the dignity of older adults with cancer who were also receiving outpatient palliative care. The findings across all racial groups also suggest that Dignity Therapy, a patient-centered approach, is promising as an intervention to improve health equity in supporting the dignity of racial minorities with cancer | - 7-item Dignity Impact Scale   Preparation for death and life completion subscales of the QUAL-E. | The loss of 22% of participants during follow-up, although within the projected dropout rate of 20% to 30%, may have unknown effects on the external validity of the findings. The sample was intentionally focused on older adults with cancer, so the findings may not be generalizable to a younger population with cancer or other potentially life-threatening conditions |
| 32 | Participants in the intervention group reported lower distress scores at 4 weeks of treatment, with small but significant differences compared to the control group. The time required to conduct the intervention ranged from 5.5 to 11 hours, and the subsequent cost depended on the compensation of the Dignity Therapy therapist | - A modified version of the Patient Distress Thermometer - Patient Dignity Inventory   Dignity therapy participant feedback questionnaire | The deterioration and death of patients during the study period affected the integrity of the data reported by the patients, which was anticipated. However, it was not possible to determine the reasons for other missing data. The impact of the sample size and missing data on the results is acknowledged, and it is not considered appropriate to generalize the findings to broader populations |
| 33 | Dignity improved for both groups at T1 (p=0.008) then decreased in both, but remained improved from baseline ‎by T2. Both groups reported low death anxiety throughout, with more in the EOL group. Death anxiety reduced in both ‎groups at T1 (p=0.033), then increased for both but remained improved from baseline by T2.‎ At baseline and T1, the EOL group reported greater social/family (p=0.079, p=0.065) and emotional wellbeing (p=0.047, ‎p=0.003) than the non-EOL group. Social/family wellbeing improved slightly for non-EOL by T2, but emotional wellbeing ‎decreased slightly for both groups.‎ The EOL group reported low posttraumatic growth (PTG) at baseline, with little impact of DT. The non-EOL group reported ‎moderate/high levels of PTG at baseline, which increased at both T1 and T2.‎ Analyses showed no significant difference in outcomes between groups, suggesting that the non-EOL group benefits from ‎DT as much as the EOL group.‎ | - | Limitations of this study include a small sample size, a non-randomized study design, and ‎short-term follow-up. |
| 34 | Dignity Therapy was effective in improving dignity-related distress, quality of life, and levels of hope. A meta-analysis found no significant differences in patients' psychological or spiritual well-being. The differences between Dignity Therapy providers in the included studies may account for the varying findings regarding the different effects of Dignity Therapy. Healthcare professionals should be trained as Dignity Therapists to enhance the visibility of Dignity Therapy for patients | - | The main limitation of this systematic review is the limited number of studies that can be meta-analyzed, due to the significant variation among the included studies. |
| 35 | Dignity Therapy produces beneficial effects at the spiritual, psycho-emotional, physical, social, and overall quality of life levels, as well as highly positive effects in alleviating the suffering of patients at the end of life, making it a useful strategy within the scope of palliative care | - | - |
| 36 | Reduction of emotional distress associated with faith. The importance of considering the patient's cultural context to understand their sense of dignity is also noted. | - | - |
| 37 | Compared to the control group, patients in the intervention group showed a significantly greater reduction in existential distress (β: -1.372, 95% CI: -2.269, -0.472; p = 0.003) and depression (β: -3.430, 95% CI: -5.032, -1.829; p < 0.001) at week one, as well as a significantly greater improvement in spiritual well-being at both week one (β: 3.705, 95% CI: 0.599, 6.811; p = 0.019) and week four (β: 4.939, 95% CI: 0.476, 9.401; p = 0.030). Family-centered Dignity Therapy has the potential to alleviate existential distress and depressive symptoms, and improve spiritual well-being. | - Demographic and clinical information Questionnaire - Patient Dignity Inventory - Patient health questionnaire-9   Functional assessment of chronic illness therapy-spiritual well-being | This study had limitations due to the relatively small sample size and a high dropout rate. Culture is an important factor that affects patients' sense of dignity, and therefore, the generalizability of the study to other cultures or countries may be limited. The outcome variables were assessed through questionnaires subjectively answered by the patients. |
| 38 | The intervention consists of three in-person sessions that facilitate participants' reminiscences and promote communication. The recruitment and response rates for the feasibility study of the intervention were 92.3% and 100%, respectively. Both patients and family caregivers reported that the intervention alleviated their psychological distress and improved communication | - | - |
| 39 | Dignity Therapy may be effective in improving hope, anxiety, and depression in adult cancer patients, but its effect on quality of life is not significant | - | There are limitations in the search strategy, as only studies published in English or Chinese were included. Therefore, some relevant studies that could influence the aggregated results may have been overlooked |
| 40 | The findings of the review show that Dignity Therapy alleviates psychological distress and improves patients' spiritual well-being and dignity. Additionally, many patients and their families found emotional support in the generativity documents created through Dignity Therapy | - | - |
| 41 | After completing Dignity Therapy (DT), a statistically significant improvement was observed in dignity-related distress scores on the Patient Dignity Inventory (PDI) (t(23) = 4.83, p < 0.001, d = 1.04) and in the dignity item (z = -3.77, p < 0.001). Anxiety (t(23) = 7.46, p < 0.001, d = 1.73), depression (t(23) = 6.08, p < 0.001, d = 0.94), emotional distress (t(23) = 9.09, p < 0.001, d = 2.45), and hopelessness (z = -4.05, p < 0.001) also showed significant improvement after the intervention. Quality of life (QoL) levels remained stable (t(23) = -0.23, p > 0.05) | - Hospital Anxiety and Depression Scale - Distress Thermometer - Patient Dignity Inventory - The European Organization for Research and Treatment of Cancer quality of life questionnaire (QLQ-30) - Patient   Feedback Questionnaire | Among the limitations of this study is its design. |
| 42 | There was a significant decrease in psychological distress scores in the experimental groups at post-test and follow-up (P ≤ 0.01). Additionally, it was found that Dignity Therapy is more useful at lower levels of the palliative performance scale | Depression-anxiety-stress scale. | - |
| 43 | Dignity therapy was the most common psychotherapy in studies where nurses were utilized as interventionists | - | - |
| 44 | Family members generally believe that Dignity Therapy helps them better prepare for the patient’s end-of-life phase and cope with the grieving process. The legacy document was considered a source of comfort, and most would recommend Dignity Therapy to others in similar situations. Dignity Therapy is generally regarded as being as important as any other aspect of the patient’s treatment | - | A key limitation of most of the studies included in this review was the small number of participants. This makes it difficult to extrapolate the conclusions to the general population |
| 45 | The results revealed the feasibility, acceptability, satisfaction, and effectiveness of Dignity Therapy for life-limiting conditions in patients across different age-related groups. The therapy was also found to be relevant in reducing anxiety, depression, and caregiver burden during the palliative care period of their loved ones | - | The review was limited to the use of Dignity Therapy in terminally ill patients. Other therapies combined with Dignity Therapy were not included. Similar studies and systematic reviews were excluded from this project |
| 46 | No statistically significant modification in the overall score or single items of the PDI were observed in the before and after analysis. The Dignity Therapy Patient Feedback Questionnaire revealed that most patients found DT helpful (82.2%) and satisfactory (92.9%). The majority (82.9%) reported that DT helped them accept the state of things, heightened their sense of dignity (78.6%) and made them feel that their life had more meaning (78.6%). 75.0% stated that DT increased their sense of self-continuity and gave them a sense of looking after unfinished business. A total of 75.0% of participants felt that DT helped their families. Nearly half of the participants reported that DT improved their quality of life and spiritual well-being (57.2%) and lessened suffering (53.6%), sadness or depression (50.0%) and the feeling of being a burden to others (46.4%). Our findings strongly support the acceptability of the intervention, but only partially support its feasibility. | - Patient Dignity Inventory   Dignity Therapy Patient Feedback Questionnaire | This is a feasibility study performed on a relatively small sample of subjects. Nevertheless, it allowed us to gather a wealth of information (both quantitative and qualitative) from both intervention users and providers.  Additionally, this was a monocentric study within a specific setting |
| 47 | Patients in the Dignity Therapy intervention maintained similar levels of peace from the pre-test and throughout the follow-up, while patients in the control group showed a decrease in peace over the same period. No significant longitudinal changes were found in measures of meaning, faith, loss of meaning and purpose, distress, coping ability, existential, psychological, or physical distress | - Karnofsky Performance Scale - FACIT-Sp - Demoralization Scale-II (DS-II)   Patient Dignity Inventory | First, the small sample size limited the study's power. Second, there was a high dropout rate, primarily due to patient deaths |
| 48 | Participants reported high levels of acceptability and effectiveness, comparable to those achieved in the face-to-face intervention. The therapist’s time was approximately 40% less, and the legacy documents were longer. Participants described online Dignity Therapy as convenient, although technological issues may pose challenges. The online delivery of Dignity Therapy is feasible and acceptable, reduces therapist time and clinical costs, and appears to reach individuals who would otherwise not receive the therapy. Email-based Dignity Therapy may have the greatest potential to reduce time and cost barriers | - Participant Feedback Questionnaire - Hospital Anxiety and Depression Scale - Herth Hope Index   FACIT-Pal | The small sample size, with an over-representation of female and educated participants, was a limitation. All participants were regular users of smartphones and/or computers. The combination of data from participants who completed the intervention via email with those who used videoconferencing may have introduced a downward bias in the therapist's time and an upward bias in the document length |
| 49 | Participants showed an increase in dignity and a reduction in demoralization and depression after receiving Dignity Therapy. Additionally, statistically significant differences were observed on day 7 (post-test 1) and day 14 (post-test 2) after the initiation of Dignity Therapy | - Dignity Inventory Mandarin Version (PDI-MV) - Demoralization Scale Mandarin Version   Patient Health Questionnaire-9 | The participants were terminal cancer patients, with the study group chosen by the patients themselves out of respect for their wishes. As a result, randomization and blinding techniques were not applied in this study |
| 50 | Patient and caregiver participants reported that Dignity Therapy was a useful tool in improving communication, allowing families to be more open about the patients’ health status. Patients reported that Dignity Therapy, as well as the open conversations prompted by it, allowed acceptance of their situation | Qualitative self-report surveys | The main limitations were the small sample size and the inability to collect data from most participants who completed the intervention, which negatively impacts the generalizability of the findings |
| 51 | An interesting aspect observed by the authors in the implementation of Dignity Therapy in pediatrics is the reluctance of parents and doctors to discuss a child's imminent death in a developmentally appropriate way.  All the children and families who participated in the process were able to complete it and expressed their gratitude for the opportunity to create the Dignity Therapy document | A novel option of Dignity Therapy by proxy was used | - |
| 52 | The original author of Dignity Therapy supported the final Portuguese version of the DT-QF-Adol, emphasizing that it captures the core dimensions of Dignity Therapy. A 100% agreement was reached on the final consensus version and the defined age group (10-18 years) | - | - |
| 53 | The results showed that Dignity Therapy led to an improvement in the quality of life in the intervention group (t 35.18 = 4.82, p = 0.001). There was also a significant difference between the two groups in terms of physical functioning (t 32.96 = -2.60, p = 0.01) and emotional functioning (t 45.69 = 6.54, p < 0.001). Additionally, Dignity Therapy was found to improve nausea and vomiting (χ2 = 5.71, p = 0.02), insomnia (χ2 = 15.78, p < 0.001), appetite (χ2 = 5.09, p = 0.02), and constipation (χ2 = 12.50, p < 0.001). | The European Organization for Research and Treatment of Cancer Quality of Life-C15-Palliative (EORTC-QLQ-C15-PAl) | The two-week interval for assessing changes in quality of life may be considered insufficient. However, this was related to the poor prognosis of the disease. Another limitation was the sampling from a single palliative care center in Tehran |
| 54 | Further research is needed to evaluate the outcomes of Dignity Therapy in terms of effectiveness and benefits for frail elderly individuals, especially those with neurocognitive disorders, as well as for caregivers | - | - |
| 55 | The primary analysis results showed higher scores for the Dignity Therapy (DT) group (mean change = 1.57) compared to the usual care (UC) group (mean change = −0.74), yielding a non-statistically significant difference in change scores of 1.44 (p = 0.670; 95% CI −5.20 to 8.06). After adjusting for baseline scores, the mean summed symptom distress score was not significant (GLM p = 0.78). The Dignity Therapy group showed a trend towards statistical improvement in anxiety (p = 0.059). The largest effects were observed in appetite improvement, reduced anxiety, and enhanced well-being, with Cohen’s effect sizes of 0.3, 0.5, and 0.31, respectively | Edmonton Symptom Scale | This study was conducted at a single private institution and does not reflect the broader scope of healthcare in Kenya, which is primarily rural and under-resourced, potentially limiting the applicability of Dignity Therapy in these contexts. A second limitation is the possible lack of statistical power, which may have prevented the primary outcome from reaching statistical significance. |
| 56 | Dignity Therapy is a beneficial intervention that has proven to be satisfactory, especially for patients with initial levels of emotional distress | - | - |
| 57 | The mean scores for hope and quality of life before the intervention were not significantly different between the experimental and control groups (P = 0.11). Four weeks after the end of the intervention, the mean hope scores in the intervention group (26.88 ± 2.90) were significantly higher than those in the control group (24.60 ± 4.26) (P = 0.03). Additionally, after the intervention, the mean quality of life scores in the intervention group (69.61 ± 12.71) were significantly higher than those in the control group (50.64 ± 12.15) (P < 0.001) | - Demographic questionnaire - The Herth-Hope questionnaire   EORTC QLQ-C30 scoring | One limitation of the study is the small sample size. Additionally, all participants were patients from a single health center |
| 58 | Dignity Therapy could be a supportive intervention to promote open conversations about life, meaning and purpose, and death between the child or young person, their family members, and healthcare professionals. However, Dignity Therapy needs to be adapted to meet their needs. Our consultation with stakeholders highlighted that Dignity Therapy has the potential to improve the psychosocial and spiritual well-being of children and young people with life-limiting or potentially fatal conditions by helping them recall memories and reflect on the things that are important to them and why they want to be remembered | - | - |
| 59 | The importance of Dignity Therapy for patients with dementia and their caregivers | - | - |
| 60 | The patient indicated that family embodied the most crucial values. He also pointed out other matters, such as his work and his lifelong passion, playing the drums. The survey questionnaire indicated that, by far, the greatest benefit was an overall improvement in the patient’s mental well-being. It also indicated benefits for the family, including hope for restoring family bonds. An unexpected therapeutic effect was the mending of a broken relationship between the patient and his daughters.  Dignity Therapy proved effective not only as a method to enhance the patient’s sense of dignity in the face of imminent death but also as a tangible tool to overcome a dramatic breakdown in communication within the family | - | - |
| 61 | The mean symptom-related distress score was significantly lower in the intervention group (mean difference = -8.60; 95% CI: -15.29 to -1.90; p = 0.022). The Dignity Therapy group showed a trend toward statistical improvement in anxiety (p = 0.059) between the two groups | - Edmonton Symptom Scale | - |
| 62 | Eight patients evaluated that the therapy positively affected their mental well-being. Dignity Therapy appears to have significant therapeutic potential, even for individuals with advanced Chronic Obstructive Pulmonary Disease (COPD) | - Hospital Anxiety and Depression Scale - Edmonton Symptom Scale - The Spiritual Needs Questionnaire (SpNQ)   Satisfaction Questionnaire | - |
| 63 | The Dignity Therapy process allowed for discussions about values related to meaning and life satisfaction, the integration and acceptance of negative experiences into the personal narrative, the reaffirmation of personal decisions and beliefs, and the adoption of new perspectives on one’s life. Participants reported higher levels of life satisfaction, a more cohesive and integrated life narrative, and improvements in self-esteem | - | - |
| 64 | The meta-analysis revealed that Dignity Therapy significantly improved dignity-related distress in the domain of existential distress (mean difference [MD]: -0.26; 95% CI, -0.50 to -0.02, p = 0.03) and in the domain of social support (MD: -0.23; 95% CI, -0.39 to -0.07, p = 0.004). A non-significant improvement was also observed in depression and anxiety. | - | Most of the Random Clinical Trials (RCTs) had a small sample size. Bias was the main methodological flaw identified in the studies included in this review. Performance bias, due to the lack of blinding of outcome assessors in certain studies, may have led to an overestimation of the effects |
| 65 | Group differences were found in the total score and in the scores for each dimension of spiritual well-being and hope level at T1 and T2 (p < 0.05). Interaction effects were statistically significant in terms of spiritual well-being (p < 0.001) and hope level (p < 0.001). The majority of patients (93.34%) and family members (96.67%) rated the Dignity Therapy intervention as positive ("very satisfactory" or "relatively satisfactory"). The implementation of Dignity Therapy in patients with hematologic malignancies in China was associated with good effectiveness in improving spiritual well-being and hope level in the short term | - Herth Hope Index - FACIT-Sp-12   The Chinese version of the European Organization for Research and Treatment of Cancer Quality of Life Questionnaire (EORTC QLQ-C30) | First, participant recruitment took place in only one province, which may limit the generalizability of the results to the entire Chinese population. Second, participants could not be blinded to Dignity Therapy. Third, the follow-up period of the study was relatively short, and long-term intervention effects were not assessed |
| 66 | Compared to standard care, Dignity Therapy decreased anxiety, depression, and dignity-related distress scores in patients with advanced cancer (SMD = -1.07; 95% CI: [-1.57, -0.58], p < 0.05; SMD = -1.31; 95% CI: [-1.92, -0.70], p < 0.05; MD = -7.30; 95% CI: [-12.04, -2.56], p < 0.05). Additionally, no significant differences were found in the patients' quality of life (QoL) (p > 0.05) | - | First, although a comprehensive search strategy was used, the current study only included reports published in English or Chinese. Second, the findings were based solely on the research included in this study. Therefore, as new related studies emerge, periodic updates of the existing results are necessary |
| 67 | Dignity Therapy is an effective tool for the treatment and improvement of patients with life-limiting conditions, especially in the final stages of life | - | - |
| 68 | It is clear that the therapy is an easy-to-use and cost-effective tool that improves the low morale of those suffering from terminal illnesses | - | - |
| 59 | The studies in the review primarily focused on the perspective of a family member and how Dignity Therapy helped their loved one, demonstrating that it was feasible and acceptable to include the family in the studies. The effects of Dignity Therapy on the family have not been well demonstrated in studies with adequate statistical power, despite the fact that serious illnesses affect not only the patient but also the family members | - | One of the main limitations of this review was the scarcity of research focusing on the effects of Dignity Therapy on the patient's family members |
| 70 | Dignity Therapy is well-received, with improvements in well-being measures. However, few studies have included young individuals (24 years or younger). In the included studies, Dignity Therapy was found to improve aspects of psychosocial well-being, emotional functioning, dignity, and hope, and was perceived as helpful for the family | - | Limitations include the restriction to articles in English, the exclusion of UK grey literature, and the omission of books, theses, and conference papers. Additionally, the reference lists of the included studies were not thoroughly searched |
| 71 | Although the DT/LP intervention did not result in improvements in the measured psychosocial outcomes, patients undergoing active treatment generally experience a deterioration in outcomes during advanced cancer and active treatment | - Distress Thermometer - Patient Dignity Inventory - FACT-Hep (Functional Assessment of Cancer Therapy-Hepatobiliary) - FACT-L (Functional Assessment of Cancer Therapy-Lung) - Linear Analogue Self-Assessment - FACIT-Sp-12   Purpose in Life Test–Short Form | The use of a small, non-diverse convenience sample, primarily from one geographic region in the United States, is a limitation. There was insufficient power to draw conclusions about the effectiveness of the combined DT/LP intervention |
| 72 | 18 patients and 24 family members rated Dignity Therapy as useful.  Dignity Therapy is feasible for German Palliative Care Units (PCUs) | - DT Patient Feedback Questionnaire - DT Family Feedback Questionnaire - Eastern Cooperative Oncology Group performance status (ECOG) - Palliative Performance Scale |  |
| 73 | Most of the questions in Dignity Talk were supported by the majority of patients and their families (>70%). Participants felt that Dignity Talk would be valuable for promoting conversations, improving connections and family relationships, enhancing the patient's sense of value and dignity, fostering effective interaction, and addressing unresolved matters. Dignity Talk can provide a gentle means to facilitate important end-of-life conversations | - | The sample size is relatively small. Participants' attitudes may not be representative of attitudes toward conversations prompted by Dignity Talk in other settings or cultural contexts |
| 74 | While Dignity Therapy is generally well-accepted in most cases, it may not always be effective, therapeutically valid, or practical, and could potentially cause family or cultural tensions | - | - |
| 75 | The results showed no significant differences between the initial and post-intervention MHI scores within each study group. Similarly, no significant differences were observed between the two study arms in MHI scores from baseline to post-intervention (baseline: mean difference = 1.54; 95% CI [-24.43 to 27.51]; p = 0.9041; post-intervention: mean difference = -12.68; 95% CI [-43.52 to 18.16]; p = 0.4046) | - Mental Health Inventory | Although this study showed no effect from the implementation of Dignity Therapy, there are important limitations. First, the study was small due to the low percentage of patients with a loved one to whom they could leave legacy documents. Second, only 55% of these loved ones agreed to participate. Another limiting factor was the high initial scores on the MHI in both the Dignity Therapy and SPC groups |
| 76 | Participants in the Dignity Therapy group showed significantly greater improvements in their sense of generativity and ego integrity compared to those in the Life Review (LR) and Waitlist Control (WC) groups.  There were no differences between the three groups in terms of dignity-related distress and perceived quality of life outcomes, including physical, social, emotional, and functional well-being. Both Dignity Therapy and Life Review interventions showed high acceptability and satisfaction. Satisfaction was also high among the family members/caregivers of Dignity Therapy participants | - The Brief Measure of Generativity and Ego-Integrity - Functional Assessment of Cancer Therapy-General, version 4 (FACT-G) - A Treatment Evaluation Form (15 items) and A Family Evaluation Form (15 items) - Palliative Care Phase Instrument, - AustraliaModified Karnofsky Performance Scale, - Resource Utilization Groupsd Activities of Daily Living,   Problem Severity Score (PSS). | Insufficient power to detect small effects due to the modest sample size in each group and the mild to moderate distress levels at the start of the study. The possibility of delayed treatment effects could not be ruled out. Participants' expectations may not have been equivalent across the three groups, given the non-blind intervention assignment, which is common in psychotherapeutic interventions |
| 77 | Of the 18 patients who completed the intervention, almost all felt that it was worthwhile, would do it again, that it met or exceeded their expectations, would recommend it to others, and mentioned that it was done at the right time. It was found that this psychosocial intervention was feasible and acceptable for cancer patients undergoing active treatment. | - Oncology Group Performance Scale score   Was It Worth It Questionnaire | The limitations of the study include a small sample size and the fact that it was conducted at a single institution. However, these limitations may be appropriate for a feasibility study |
| 78 | Dignity Therapy was associated with a significant decrease in Demoralization Syndrome (DS) compared to Standard Palliative Care (SPC).  Similarly, Dignity Therapy was associated with a significant reduction in the prevalence of Desire for Death (DfD). Compared to participants assigned to the control group, those who received Dignity Therapy showed a statistically significant reduction in 19 out of 25 items on the Patient Dignity Inventory (PDI).  Dignity Therapy had a beneficial effect on the psychological distress faced by patients near the end of life. | - Desire for Death Rating Scale (DDRS)   Patient Dignity Inventory | The sample was primarily composed of older patients. As such, the influence of Dignity Therapy on younger cohorts remains uncertain and warrants further investigation |
| 79 | After the treatment, the patient and his wife showed improvements in measures of meaning, mutuality, and caregiver and recipient burden, respectively. The patient reported reduced symptoms of depression, while his wife reported greater life satisfaction. Both reported high satisfaction with the treatment, increased willingness to discuss distress, greater acceptance of health changes, and better adaptability to those changes | - | - |
| 80 | After the intervention, there was a reduction in depression levels (from 40 to 29 points) and an improvement in the patient’s sense of dignity (from 104 to 76 points), although anxiety levels increased (from 46 to 53 points). In addition, it is noted that the Dignity Therapy technique facilitated the recovery of positive memories, the possibility of regret and asking for forgiveness, and the strengthening of family bonds at the end of life | - Beck Anxiety Inventory, - Beck Depression Inventory,   Patient Dignity Inventory | - |
| 81 | The repeated measures t-test results showed statistically significant differences in dignity, anxiety, spirituality, and quality of life dimensions for both groups. However, depression increased in the Dignity Therapy group after the intervention, and no differences were found regarding resilience. Therapy in the counseling group did not negatively affect depression, and resilience improved. When post-intervention differences between groups were calculated, statistically significant differences were found in anxiety, with lower scores in the counseling group (t(68) = -2.341, p = 0.022, d = 0.560). | - Patient Dignity Inventory - Hospital Anxiety and Depression Scale - Brief Resilient Coping Scale (BRCS) - GES Questionnaire, the Duke-UNC-11 Functional Social Support Questionnaire   Questionnaire (EORTC-QLQ-C30). | The use of an inclusion criterion related to dignity (patients' interest in dignity) may have introduced some bias, limiting the generalizability of the results. Additionally, although supervised by the research team, the randomization process, interventions, and data collection were carried out by the same professional, making it difficult to assess treatment adherence and fidelity |
| 82 | The study demonstrated that Dignity Therapy is feasible, acceptable, and potentially effective for older adults with dementia.  As a psychosocial intervention, Dignity Therapy has the potential to improve quality of life and enhance person-centered care for individuals with dementia | - The Herth Hope Index (HHI) - Patient Dignity Inventory - Perceived Quality of Life, and Satisfaction with Quality Life Ratings | The findings are limited by scope and size. The study was not aimed at evaluating the effectiveness of Dignity Therapy, although it provides useful indicators regarding the suitability of outcome measures for future studies |
